# Cognitive impairments in a Down syndrome model with abnormal hippocampal and prefrontal dynamics and cytoarchitecture

**DOI:** 10.1016/j.isci.2023.106073

**Published:** 2023-01-28

**Authors:** Phillip M. Muza, Daniel Bush, Marta Pérez-González, Ines Zouhair, Karen Cleverley, Miriam L. Sopena, Rifdat Aoidi, Steven J. West, Mark Good, Victor L.J. Tybulewicz, Matthew C. Walker, Elizabeth M.C. Fisher, Pishan Chang

**Affiliations:** 1 Department of Neuromuscular Diseases, UCL Queen Square Institute of Neurology, London WC1N 3BG, UK; 2 Department of Clinical and Experimental Epilepsy, UCL Queen Square Institute of Neurology, London WC1N 3BG, UK; 3 UCL Institute of Cognitive Neuroscience and UCL Queen Square Institute of Neurology, University College London, London WC1N 3AZ, UK; 4 Bioinformatics and Biostatistics, The Francis Crick Institute, 1 Midland Road, London NW1 1AT, UK; 5 Immune Cell Biology and Down Syndrome Laboratory, The Francis Crick Institute, London NW1 1AT, UK; 6 Sainsbury Wellcome Centre, University College London, London W1T 4JG, UK; 7 School of Psychology, Cardiff University, Cardiff CF10 3AT, UK; 8 School of Physiology, Pharmacology, and Neuroscience, University of Bristol, Bristol BS8 1TD, UK

**Keywords:** Developmental neuroscience, Transcriptomics, Model organism

## Abstract

The Dp(10)2Yey mouse carries a ∼2.3-Mb intra-chromosomal duplication of mouse chromosome 10 (Mmu10) that has homology to human chromosome 21, making it an essential model for aspects of Down syndrome (DS, trisomy 21). In this study, we investigated neuronal dysfunction in the Dp(10)2Yey mouse and report spatial memory impairment and anxiety-like behavior alongside altered neural activity in the medial prefrontal cortex (mPFC) and hippocampus (HPC). Specifically, Dp(10)2Yey mice showed impaired spatial alternation associated with increased sharp-wave ripple activity in mPFC during a period of memory consolidation, and reduced mobility in a novel environment accompanied by reduced theta-gamma phase-amplitude coupling in HPC. Finally, we found alterations in the number of interneuron subtypes in mPFC and HPC that may contribute to the observed phenotypes and highlight potential approaches to ameliorate the effects of human trisomy 21.

## Highlights

• Dp(10)2Yey mice have impaired spatial memory function
• Dp(10)2Yey mice show increased anxiety-like behavior
• These deficits are associated with altered neural activity in the mPFC and HPC
• This may be explained by altered interneuron subtype distribution in mPFC and HPC

## Graphical abstract

**Figure:**
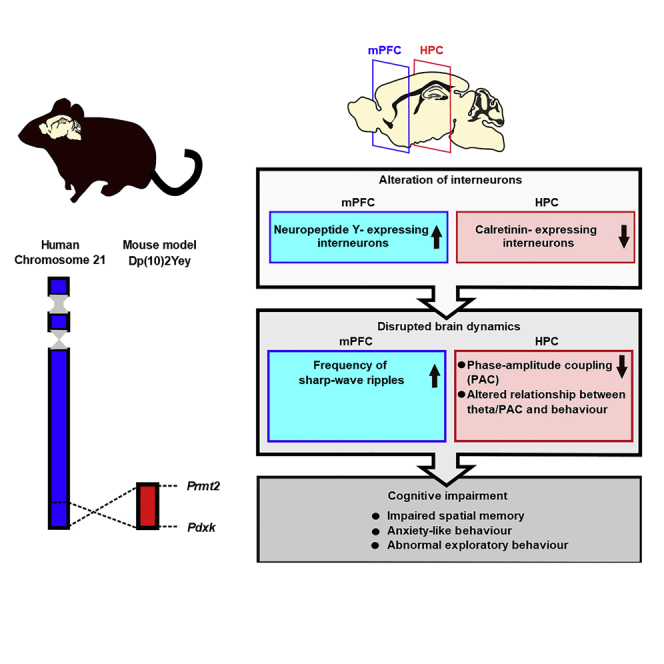

## Introduction

Down syndrome (DS) is a complex chromosomal disorder arising from trisomy of human chromosome 21 (Hsa21). DS has an incidence of ∼1 in 800 live births worldwide,^1^ and the current global population of people with DS is estimated at 6 million.^2^ The prevalence of DS is rising, primarily due to increased maternal age and increased life expectancy in people with DS resulting from reduced infant mortality rates and improved access to health care.^3^^,^^4^ A majority of individuals with DS have mild to severe intellectual dysfunction.^5^^,^^6^ In addition, individuals with DS commonly exhibit depressed mood, anxiety, decreased interest, and slowed psychomotor behaviors.^7^^,^^8^^,^^9^^,^^10^^,^^11^

Animal models give valuable insights into gene dosage imbalances in DS.^12^^,^^13^ In particular, mouse models of DS have been crucial to investigate the links between dosage-sensitive “DS genes” and phenotypes and have provided the foundation for potential treatments for DS comorbidities.^13^^,^^14^ Hsa21 has synteny with a large stretch of mouse chromosome 16 (Mmu16; approximately 145 protein-coding genes) and shorter regions of Mmu10 (37 protein-coding genes) and Mmu17 (19 protein-coding genes).^12^^,^^15^ Chromosome engineering has enabled the creation of specific mouse models with intra-chromosomal duplications, to investigate genotype-phenotype relationships in DS; for example, such models have duplications of entire syntenic segments of Mmu16 (Dp(16)1Yey and Dp1Tyb models), Mmu17 (Dp(17)3Yey), or Mmu10 (Dp(10)2Yey).^13^

Previously, we have described altered neural dynamics in the hippocampus (HPC) and medial prefrontal cortex (mPFC) alongside distinct cognitive impairments in these different DS models.^16^ These regions play a fundamental role in executive function and emotional regulation,^17^^,^^18^^,^^19^ mnemonic processing,^20^ planning, and decision-making.^21^^,^^22^^,^^23^^,^^24^^,^^25^ Functional connectivity between these regions is implicated in spatial memory function,^26^^,^^27^ modulation of anxiety,^28^^,^^29^ and fear.^30^^,^^31^ Thus, alterations in neuronal oscillations within these regions could underlie the cognitive and emotional dysfunction observed in DS.

The Dp(10)2Yey mouse model of DS carries a small internal duplication spanning only 37 Hsa21 protein-coding orthologs mapping to Mmu10.^32^ Several genes in the Mmu10 region, including *Adarb1*, *S100b*, *Cstb*, *Prmt2*, and *Trpm2*, play a crucial role in neurodevelopment and brain function, but it is unknown if these genes are dosage sensitive and give rise to phenotypes when present in three copies.^32^^,^^33^^,^^34^^,^^35^^,^^36^ Aberrant dosage of any of these genes—or others in the Dp(10)2Yey duplication—could contribute to impairments seen in the mouse and human brain. Previously, we showed that Dp(10)2Yey DS mice exhibit impaired spatial memory function and reduced HPC theta-gamma coupling.^16^

Here, we use a combination of behavioral, electrophysiological, and histological analyses to further characterize DS-related phenotypes, and assess alterations in neural activity and cell expression in Dp(10)2Yey mice. We confirm and extend the observation that these animals show impairments in spatial memory function, and we also demonstrate increased anxiety-like behavior. Crucially, these deficits are associated with an increased incidence of sharp-wave ripples (SWRs) in mPFC during memory consolidation and reduced theta-gamma phase-amplitude coupling (PAC) in HPC during exploration of a novel environment. Furthermore, we show that these animals have altered expression of specific interneurons in mPFC (increased neuropeptide-Y [NPY]-expressing interneurons) and HPC (decreased calretinin [CR]-expressing interneurons), which likely contributes to the observed abnormalities in neural oscillations and DS-associated behavior. These findings will facilitate better understanding of the mechanisms underlying developmental cognitive disability and increased anxiety in DS and pave the way for the determination of key dosage-sensitive genes within the Dp(10)2Yey region of duplication. Such genes and their products/pathways are potential targets for new therapies to help ameliorate behavioral dysfunction in DS.

## Results

### Impaired spatial memory and increased rate of mPFC SWRs during memory consolidation in Dp(10)2Yey mice

**Figure 1:**
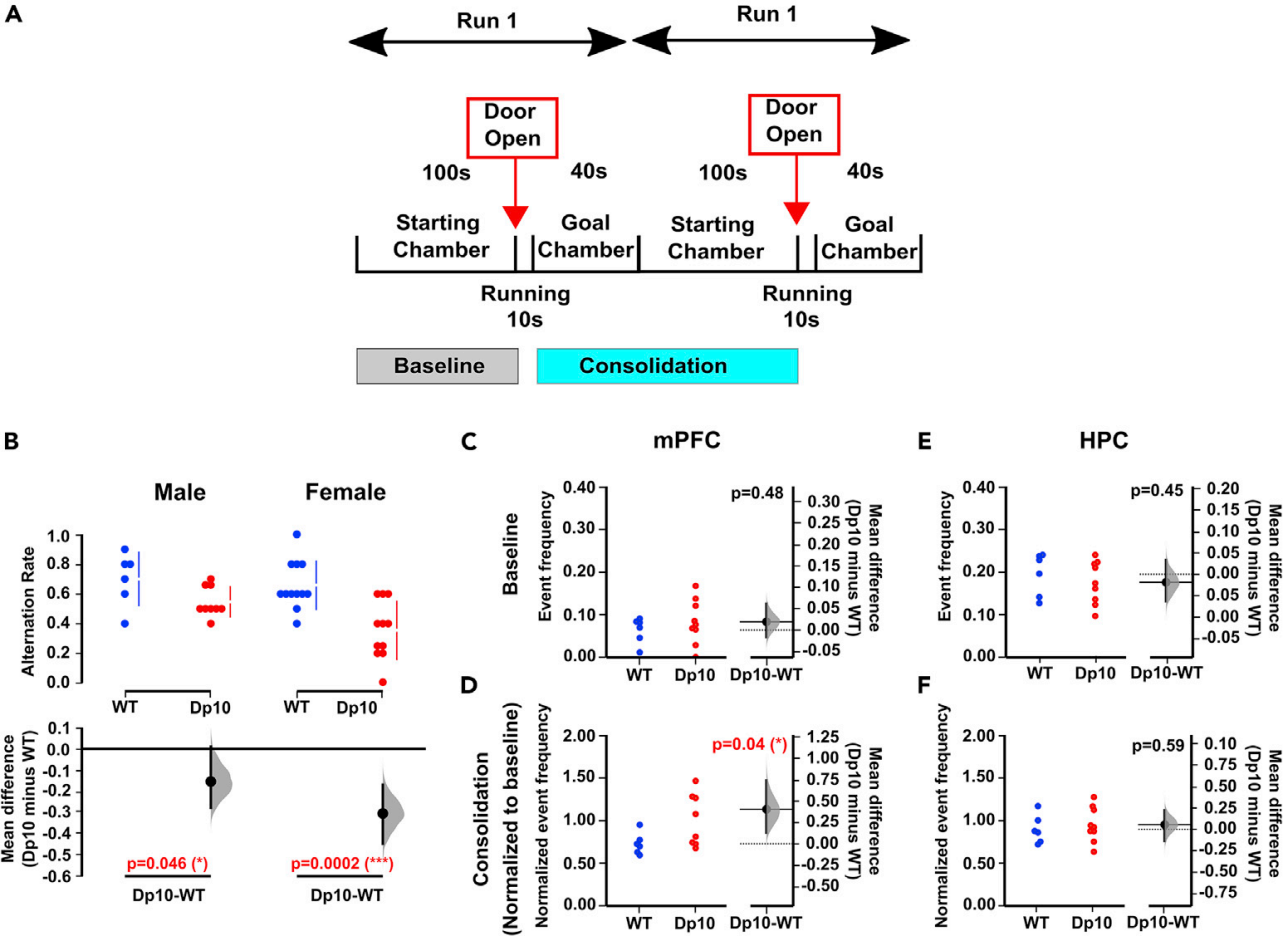
Reduced spontaneous alternation and increased rate of mPFC SWRs during consolidation in Dp(10)2Yey mice (A) Schematic of the experimental procedure. (B) Comparison of alternation rate in male (n = 9) and female (n = 11) Dp(10)2Yey mice (Dp10, red) at age 3–4 months compared with their wild-type control littermates at age 3–4 months (WT, blue, n = 6 male and n = 12 female). Upper panel shows a scatterplot of raw data from individual animals, with a line indicating the 90% confidence intervals; lower panel shows the bootstrap sampling distributions of paired mean differences in Cumming estimation plots. The mean differences are depicted as black dots, and the black line shows the 95% confidence interval. (C–F) The incidence rate of SWRs in the (C and D) mPFC and (E and F) HPC during the (C and E) baseline (event frequency, Hz) and (D and F) consolidation periods (change in event frequency, normalized to baseline). Left panel shows a scatterplot of raw data from individual animals; right panel shows the bootstrap sampling distribution of paired mean differences in Gardner-Altman estimation plots. The mean differences are depicted as black dots, and the black line shows the 95% confidence interval. Statistical analysis was performed using a permutation test (with 5000 shuffles). All statistical details are presented in Data S1.

Previously, we have shown that male Dp(10)2Yey mice exhibit impaired spatial memory in a spontaneous alternation task.^16^ Here, our first aim was to determine whether this phenotype was specific to male mice or common across sexes. Hence, we repeated this behavioral task with a female cohort of Dp(10)2Yey DS mice and wild-type (WT) control littermates. Consistent with our previous study,^16^ we found that both male and female Dp(10)2Yey mice exhibited significantly lower alternation rates compared with WT (Figures 1A and 1B). This suggests that there are no sex differences in spatial memory function in this DS mouse model. Interestingly, we found that alternation rate was significantly lower than chance level (0.5) in female (Wilcoxon signed-rank test, W = −2.03, p = 0.04), but not male (Wilcoxon signed-rank test, W = 0.96, p = 0.33) Dp(10)2Yey mice. This might suggest that the female mice were reluctant to explore the unknown, novel arm because of increased anxiety. In contrast, both WT cohorts exhibited an alternation rate that was significantly higher than chance (Wilcoxon signed-rank test, male WT: W = 1.99, p = 0.04; female: W = 2.65, p = 0.01).

Growing evidence suggests that SWRs in the HPC and mPFC^37^^,^^38^ support memory consolidation and memory-guided behavior.^39^ Hence, we next examined SWR activity in both the HPC and mPFC of male Dp(10)2Yey animals during rest periods in the spontaneous alternation task (see Figure S1 for representative traces). We found that there were no significant differences in the incidence rate of SWRs in either mPFC (Figure 1C) or HPC (Figure 1D) during the baseline period before their first run on the T-maze, compared with WT. Conversely, SWRs occurred significantly more often in the mPFC (Figure 1E) but not HPC (Figure 1F) of Dp(10)2Yey mice during the consolidation period between the first and second runs on the T-maze, compared with WT. However, there was no significant difference in either the ripple-band amplitude or duration of these SWR events between groups (Figure S2). This suggests that impaired spatial alternation performance in this DS mouse model is associated with an increased incidence of mPFC SWRs during memory consolidation. Importantly, this difference was not observed in other DS mouse models (Figure S3), suggesting that it is a phenotype arising specifically from genes within the duplicated region of Mmu10.

### Dp(10)2Yey mice exhibit anxiety-like behavior in a novel environment

**Figure 2:**
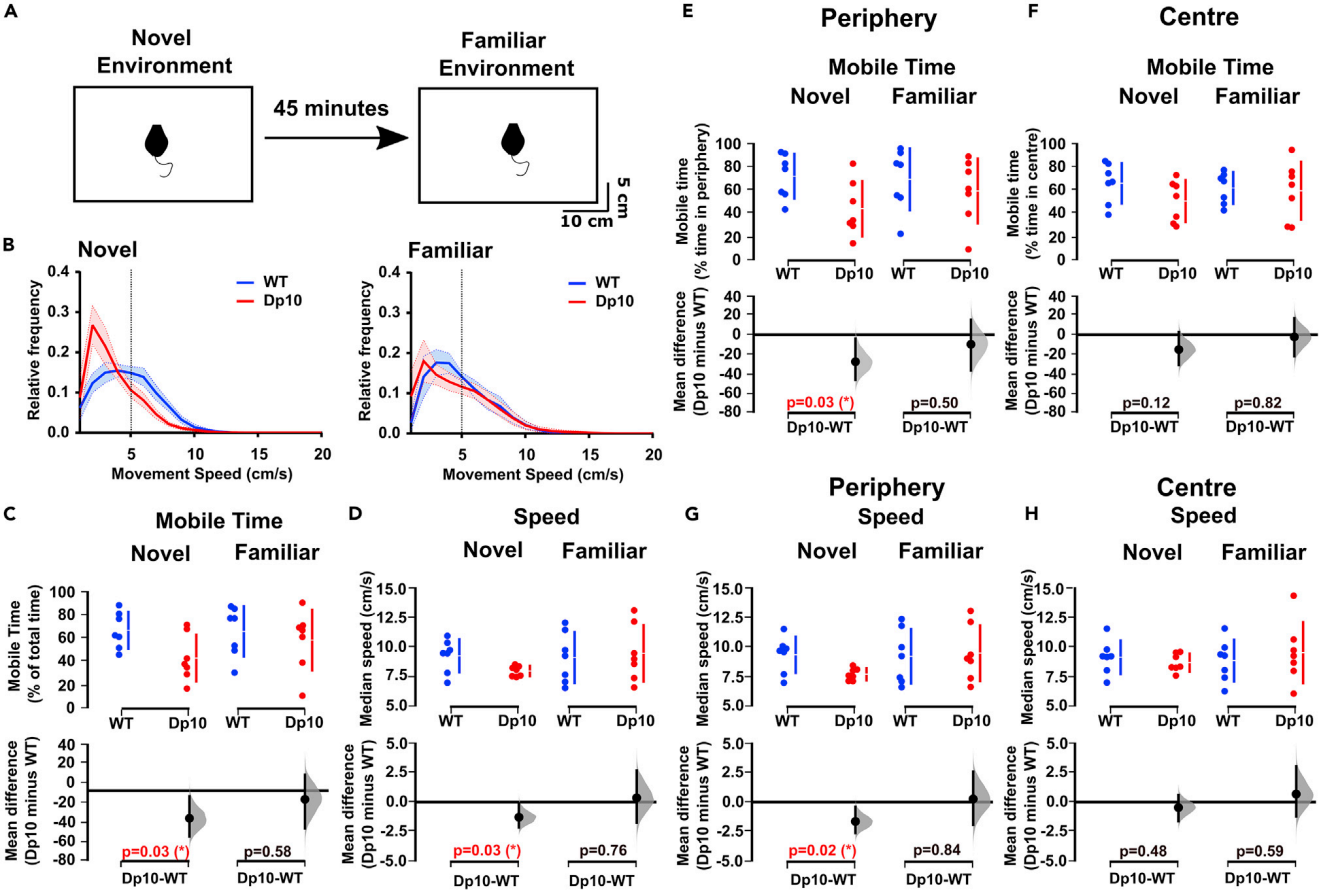
Dp(10)2Yey mice show reduced mobility in a novel open field environment (A) Schematic of the experimental procedure. (B) Relative frequency of movement speeds in a novel and familiar environment for Dp(10)2Yey mice at age 3–4 months (Dp10, red, n = 7) and wild-type control littermates at age 3–4 months (WT, blue, n = 7). Each line shows the grand-average relative frequency of movement speed across animals; shaded area indicates the SEM mean; vertical dotted line indicates the threshold between movement and stationary periods (5 cm/s). (C and D) Comparison of (C) time spent mobile (i.e., >5 cm/s, as a percentage of total time) and (D) median running speed during movement (cm/s) between Dp(10)2Yey mice and wild-type control littermates in a novel and familiar environment. (E and F) Comparison of time spent mobile while in the (E) periphery (as a percentage of total time spent in the periphery) and (F) center (as a percentage of total time spent in the center) between Dp(10)2Yey and WT littermates in a novel and familiar environment. (G and H) Comparison of median running speed during movement while in the (G) periphery and (H) center between Dp(10)2Yey and WT littermates in a novel and familiar environment. For (C–H) the upper panel shows a scatterplot of raw data from individual animals, with a line indicating the 90% confidence intervals, and the lower panel shows the bootstrap sampling distribution of paired mean differences in Cumming estimation plots. The mean differences are depicted as black dots, and the black line shows the 95% confidence interval. Statistical analysis was performed using a permutation test (with 5000 shuffles). All statistical details are presented in Data S1.

DS is associated with mild to severe cognitive disability and depressed mood, anxiety, decreased interest, and slowing psychomotor behaviors compared with typically developing individuals.^7^^,^^8^^,^^9^^,^^10^^,^^11^ In addition, functional interactions between the HPC and mPFC are implicated in anxiety-like behavior in rodents.^28^ Therefore, we asked whether dysfunction within this circuitry was associated with increased anxiety in Dp(10)2Yey mice. To do so, we recorded behavior and concurrent local field potential (LFP) signals from these regions during repeated visits to an open field environment (Figure 2A).

First, focusing on behavior, we found that Dp(10)2Yey mice exhibit altered movement statistics in novel, but not familiar, open field environments compared with WT littermates (Figures 2B and S4). Specifically, male Dp(10)2Yey mice spent less time mobile (Figure 2C) and ran more slowly when they were mobile (Figure 2D) in novel, than in familiar, environments. Interestingly, these effects appeared to be specific to the periphery of the open field (i.e., the half of the environment closest to the walls), with Dp(10)2Yey mice spending less time mobile (Figures 2E and 2F) and running more slowly when mobile (Figures 2G and 2H) in this area, but not in the center of the open field. This suggests that these mice experience increased anxiety in novel environments, compared with WT animals, which manifests as a reluctance to explore the open field. Consistent with this observation, in a separate set of purely behavioral experiments, we observed significantly fewer climbing bouts in male Dp(10)2Yey mice during an elevated-platform task (Figure S5). Importantly, there were no differences in anxiety-like behavior in the Dp1Tyb model compared with WT littermates in a novel environment (Figure S6), indicating strain-specific behavior in Dp(10)2Yey animals that likely arises from specific Mmu10 dosage-sensitive genes.

### HPC theta power correlates with exploratory behavior in novel but not familiar environments

**Figure 3:**
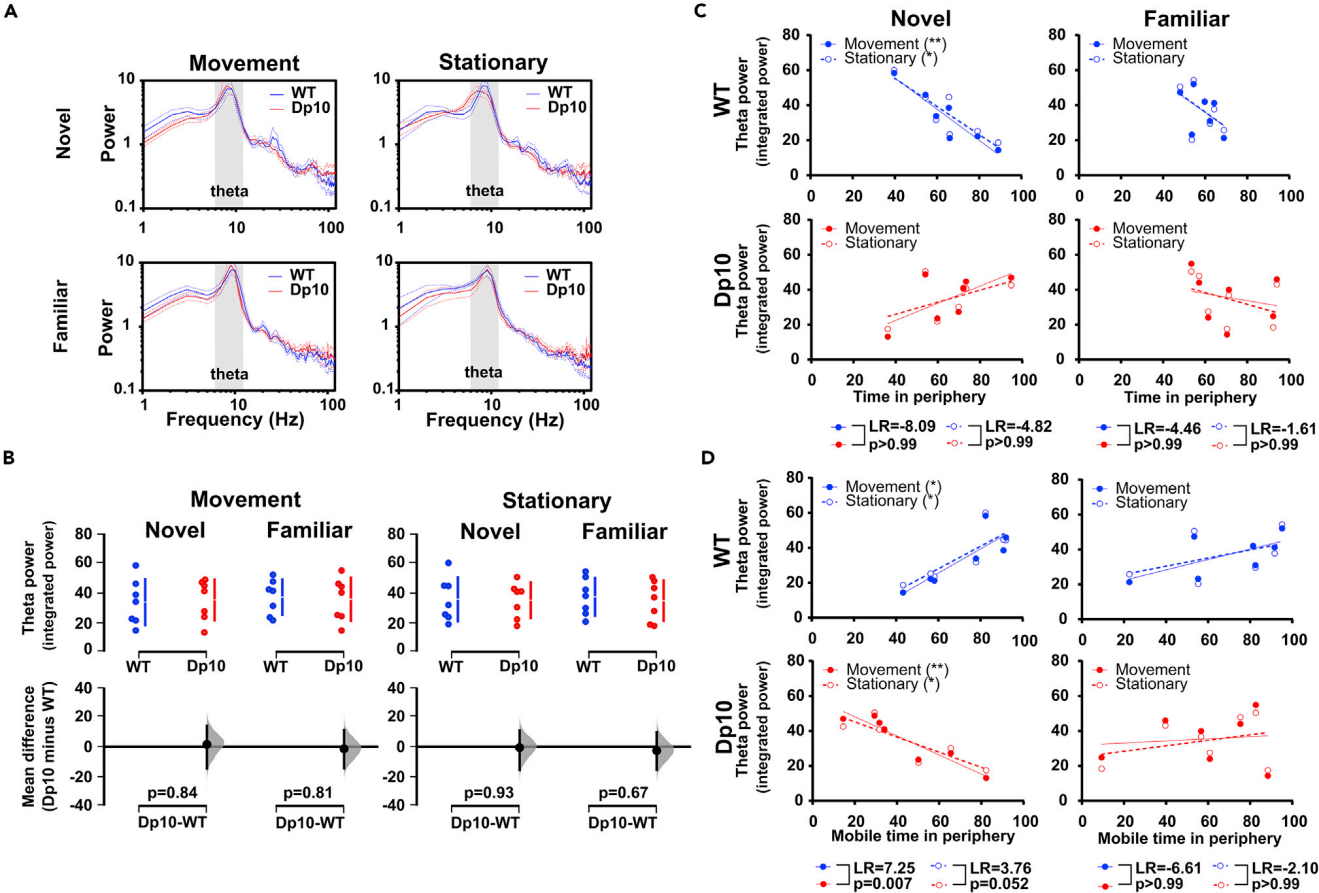
HPC theta power in novel and familiar environments (A) Hippocampal LFP power spectra in novel and familiar environments. Each line shows the grand-average spectra for Dp(10)2Yey at age 3–4 months (Dp10, red, n = 7) and WT control littermates at age 3–4 months (WT, blue, n = 7), with the dotted line representing the SEM. (B) Comparison of integrated theta power during movement and stationary periods in a novel and familiar environment. The upper panel shows a scatterplot of raw data from individual animals, with a line indicating the 90% confidence intervals, and the lower panel shows the bootstrap sampling distribution of paired mean differences in Cumming estimation plots. The mean differences are depicted as black dots, and the black line shows the 95% confidence interval. Statistical analysis was performed using a permutation test (with 5000 shuffles). (C and D) Relationship between integrated theta power and behavior across animals. (C) Proportion of time spent in the periphery and (D) proportion of time spent mobile while in the periphery. Continuous line shows the linear regression. The Pearson correlation test was used to measure a linear dependence, ∗p < 0.05, ∗∗p < 0.01. Pearson correlation coefficients (R) and significance (p values) are presented in Table S1. The likelihood ratio (LR) test was used to compare regression models. All statistical details are presented in Data S1.

Next, we investigated theta band activity in the LFP signal recorded from the HPC and mPFC of Dp(10)2Yey and WT mice during the open field test. We found no differences in theta power between groups in either the HPC (Figures 3A and 3B) or mPFC (Figures S7A and S7B) during movement or stationary periods in either novel or familiar environments. In addition, there were no significant differences in peak theta frequency between groups (repeated measures ANOVA, HP_Movement_: F_(1, 12)_ = 0.26; HP _stationary_: F_(1, 12)_ = 0.10; mPFC_Movement_: F _(1, 12)_ = 0.02; mPFC_Stationary_: F_(1, 12)_ = 0.01, all p > 0.62). This is consistent with previous results from the same DS mouse model during the spontaneous alternation task described above.^16^

Intriguingly, hippocampal theta power in WT mice was significantly correlated with exploratory behavior in novel but not familiar environments. Specifically, animals with greater HPC theta power tended to spend less time in the periphery of the open field (Figure 3C) and a greater proportion of time mobile while they were in the periphery (Figure 3D). Conversely, Dp(10)2Yey mice showed no relationship between theta power and time spent in the periphery of the open field, and a significant negative correlation between theta power and the proportion of time spent mobile while in the periphery (i.e., animals with greater HPC theta power spent a greater proportion of time stationary while they were in the periphery). A comparison of regression models between Dp(10)2Yey and WT mice using a likelihood ratio test indicated that the slope of this latter relationship was significantly different between groups (Figure 3D). In sum, these results describe an opposing relationship between hippocampal theta power and exploratory behavior while in the periphery of a novel environment. In contrast, we did not see any relationship between mPFC theta power and movement statistics in either Dp(10)2Yey mice or WT littermates (Figures S7C and S7D).

### Dp(10)2Yey mice show reduced HPC phase-amplitude coupling in a novel environment

**Figure 4:**
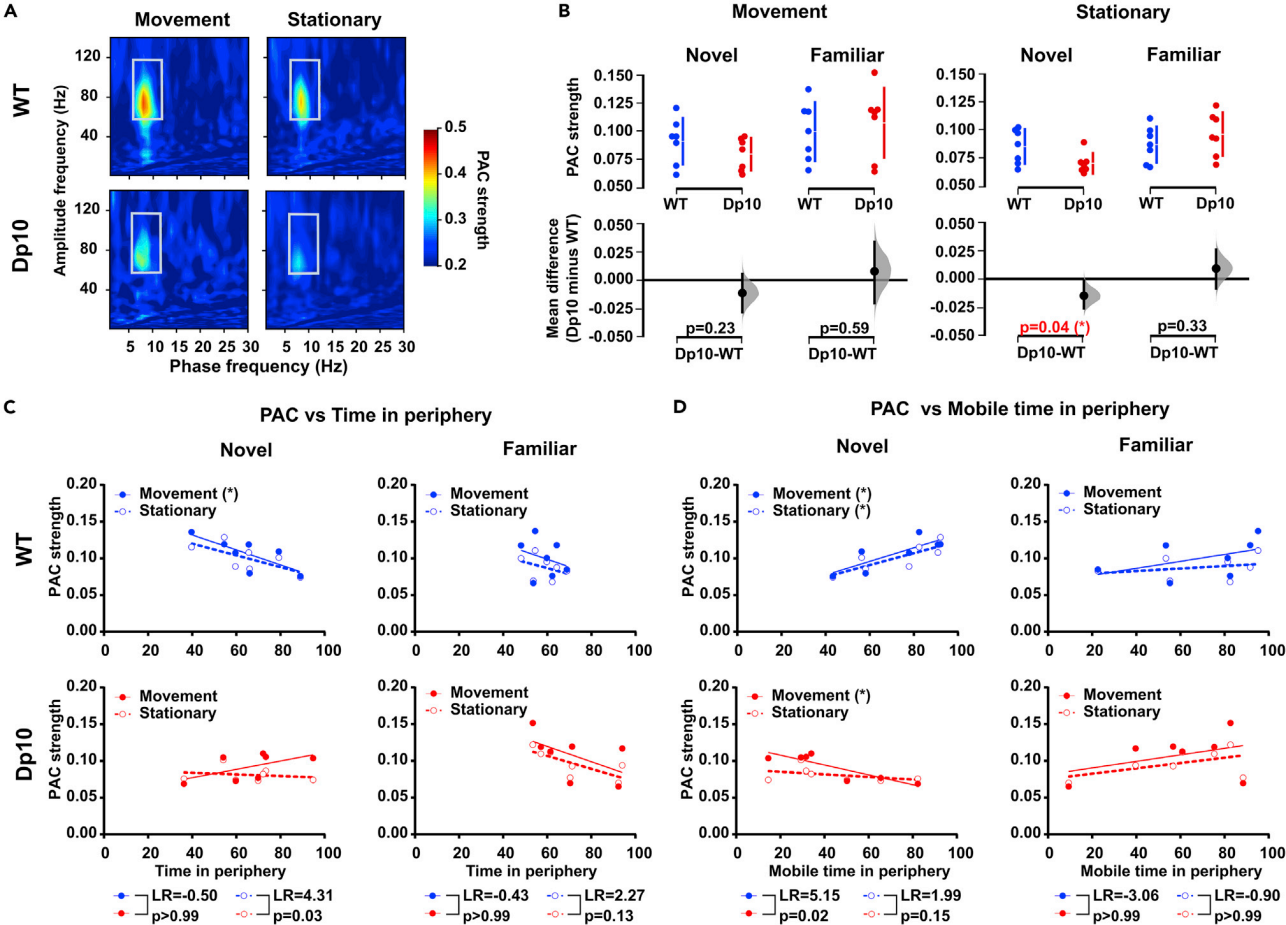
Dp(10)2Yey mice show reduced HPC phase-amplitude coupling in novel environments (A) Comodulograms showing the magnitude of HPC phase-amplitude coupling across a range of low- (phase) and high- (amplitude) frequency bands in n = 7 Dp(10)2Yey and n = 7 WT control littermates (all at age 3–4 months) during movement and stationary periods in a novel environment. White box indicates the 6–12 Hz theta phase and 60–120 Hz gamma amplitude window on which we focused in these analyses. (B) Comparison of theta-gamma PAC during movement and stationary periods between Dp(10)2Yey (Dp10, red) and WT control littermates (WT, blue) mice in novel and familiar environments. The upper panel shows a scatterplot of raw data from individual animals, with a line indicating the 90% confidence intervals, and the lower panel shows the bootstrap sampling distribution of paired mean differences in Cumming estimation plots. The mean differences are depicted as black dots, and the black line shows the 95% confidence interval. Statistical analysis was performed using a permutation test (with 5000 shuffles). (C and D) Relationship between theta-gamma PAC and behavior across animals. (C) Proportion of time spent in the periphery and (D) proportion of time spent mobile while in the periphery. Continuous line shows the linear regression. The Pearson correlation test was used to measure a linear dependence, ∗p < 0.05, ∗∗p < 0.01. Pearson correlation coefficients (R) and significance (p values) are presented in Table S1. The likelihood ratio (LR) test was used to compare regression models. All statistical details are presented in Data S1.

Cross-frequency PAC of neuronal oscillations has been proposed to be a general mechanism used by the brain to perform the network-level dynamic computations that underlie voluntary behavior.^40^ Indeed, theta-gamma PAC in the HPC is thought to modulate exploratory and memory-guided behaviors.^41^^,^^42^^,^^43^ Previously, we showed that Dp(10)2Yey mice exhibit weaker theta-gamma PAC in the HPC during spontaneous alternation.^16^ We therefore asked whether aberrant theta-gamma PAC is also observed in the open field, and whether it is associated with the altered exploratory behavior observed in Dp(10)2Yey mice. Interestingly, we found that theta-gamma PAC was weaker in Dp(10)2Yey mice than WT littermates in novel, but not familiar, environments (Figures 4A and 4B). Moreover, this effect appears to be primarily driven by differences in theta-gamma PAC during stationary periods. These results indicate that reduced theta-gamma PAC in the HPC of Dp(10)2Yey mice is not specific to tasks with an explicit spatial memory component.

Next, we asked whether the strength of theta-gamma PAC correlated with exploratory behavior in novel environments. As expected, and similar to the relationships between integrated theta power and movement statistics described above, we found that hippocampal theta-gamma PAC in WT mice was significantly correlated with exploratory behavior in novel, but not familiar, environments. Specifically, animals with stronger modulation of HPC gamma power by theta phase during movement tended to spend less time in the periphery of the open field (Figure 4C); and those with stronger theta-gamma PAC during movement or stationary periods spent a greater proportion of time mobile while they were in the periphery (Figure 4D). Conversely, Dp(10)2Yey mice showed no relationship between theta-gamma PAC and time spent in the periphery of the open field, and a significant negative correlation between theta-gamma PAC during movement and the proportion of time spent mobile while in the periphery (i.e., animals with greater HPC theta-gamma PAC during movement spent a greater proportion of time stationary while they were in the periphery). A comparison of regression models between Dp(10)2Yey and WT mice using a likelihood ratio test indicated that the slope of this latter relationship was significantly different between groups (Figures 4C and 4D).

### A subtype-specific alteration of interneurons is evident in Dp(10)2Yey mPFC and HPC

Cortical circuits incorporating GABAergic interneurons controlling spike timing in individual neurons can synchronize network activity and further contribute to network functions.^44^ Altered GABAergic activity correlates with abnormal theta and gamma oscillations,^45^ as well as reductions in sociability, cognitive impairments, and alterations in anxiety-related behaviors in humans and in animals.^46^^,^^47^^,^^48^ To investigate the mechanism(s) underlying abnormal rhythmic activity in the Dp(10)2Yey brain, we assessed mPFC and HPC GABAergic interneurons and hippocampal and synaptic function and dendritic spine density.

**Figure 5:**
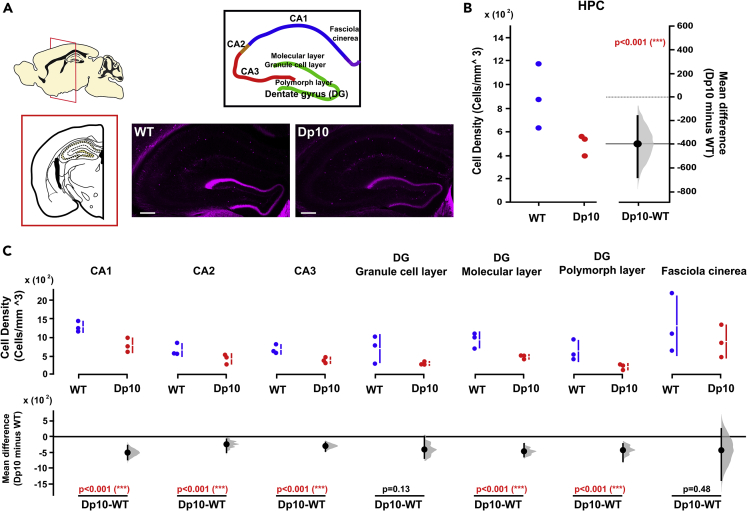
Reduced numbers of calretinin (CR)-expressing interneurons in the HPC of Dp(10)2Yey DS mice (A) Mouse brain atlases highlighting the regions of interest: HPC CA1 region (CA1), HPC CA3 region (CA3), dentate gyrus (DG) with three distinct layers (molecular layer, granule cell layer, and polymorphic layer), and fasciola cinerea. Representative pictures of staining in the HPC of WT and Dp10 mice. Scale bar, 200 μm. (B) Number of CR-expressing interneurons in HPC (cell density; cells/mm^3^) was significantly lower in n = 3 Dp(10)2Yey than n = 3 WT littermates (at age 3–4 months). Left panel shows a scatterplot of raw data from individual animals; right panel shows the bootstrap sampling distribution of paired mean differences in Gardner-Altman estimation plots. The mean differences are depicted as black dots, and the black line shows the 95% confidence interval. (C) CR-expressing interneurons in different HPC subregions of Dp(10)2Yey and WT mice. The upper panel shows a scatterplot of raw data from individual animals, with a line indicating the 90% confidence intervals, and the lower panel shows the bootstrap sampling distribution of paired mean differences in Cumming estimation plots. The mean differences are depicted as black dots, and the black line shows the 95% confidence interval. Statistical analysis was performed using a permutation test (with 5000 shuffles). All statistical details are presented in Data S1.

**Figure 6:**
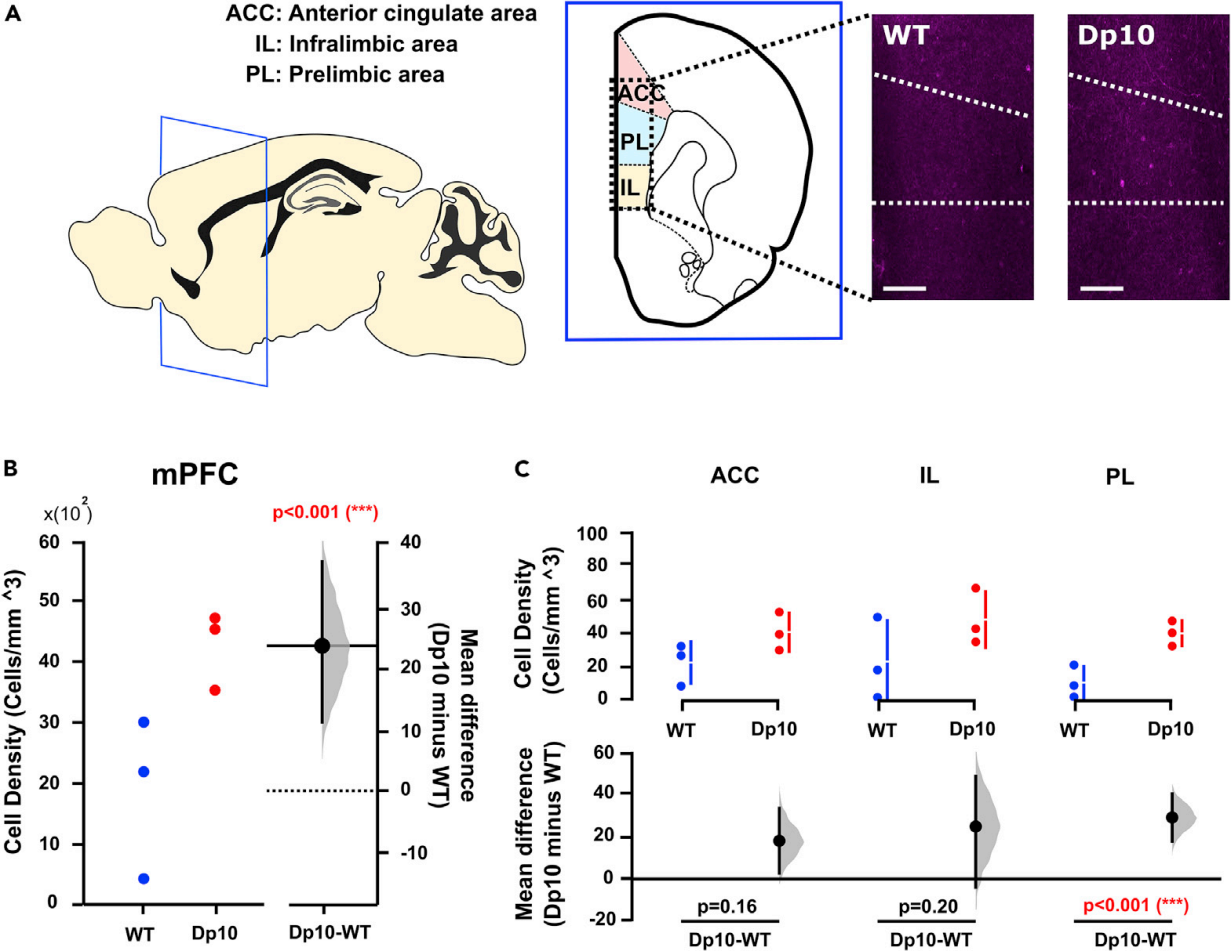
Increased numbers of neuropeptide Y (NPY)-expressing interneurons in the mPFC of Dp(10)2Yey mice (A) Mouse brain atlases highlighting the regions of interest: mPFC area includes anterior cingulate, prelimbic cortex, and infralimbic cortex, and representative pictures of staining in the mPFC of WT and Dp10 mice. Scale bar, 200 μm. (B) Number of NPY-expressing interneurons in mPFC (cell density; cells/mm^3^) was significantly higher in n = 3 Dp(10)2Yey mice than n = 3 WT littermates (at age 3–4 months). Left panel shows a scatterplot of raw data from individual animals; right panel shows the bootstrap sampling distribution of paired mean differences in Gardner-Altman estimation plots. The mean differences are depicted as black dots, and the black line shows the 95% confidence interval. (C) NPY-expressing interneurons in the different mPFC subregions in Dp(10)2Yey and WT mice. The upper panel shows a scatterplot of raw data from individual animals, with a line indicating the 90% confidence intervals, and the lower panel shows the bootstrap sampling distribution of paired mean differences in Cumming estimation plots. The mean differences are depicted as black dots, and the black line shows the 95% confidence interval. Statistical analysis was performed using a permutation test (with 5000 shuffles). All statistical details are presented in Data S1.

We found that the density of CR-, but not parvalbumin (PV)- and NPY-, expressing interneurons was significantly lower in the HPC in Dp(10)2Yey than WT littermates (Figures 5A, 5B, S8, S9 and Table S2). Interestingly, we found that the changes in HPC CR-expressing interneurons were region specific: significantly fewer CR-expressing interneurons were observed in CA1, CA2, and CA3, as well as the molecular and polymorphic layers of the dentate gyrus, but we saw no differences in the dentate gyrus granule cell layer or fasciola cinerea (Figures 5A, 5C, S8, and S9). Conversely, the density of NPY-expressing interneurons was significantly higher in mPFC of Dp(10)2Yey mice (Figures 6A and 6B), but we observed no differences in CR- or PV-expressing interneurons (Table S2). Again, we found region-specific alterations: the density of NPY-expressing interneurons was significantly higher in the prelimbic cortex, but not in anterior cingulate or infralimbic cortex (Figures 6A and 6C).

Abnormal dendritic spine structure and function are seen in postmortem DS brain, including reductions in spine density and abnormal spine morphology.^49^^,^^50^^,^^51^ Hence, we next investigated synaptic function and dendritic spine density in Dp(10)2Yey mice using immunoblotting and Golgi-Cox staining. However, we did not find significant changes in the synaptic markers analyzed (Figure S10A) or in dendritic spine density (Figure S10B) in HPC of Dp(10)2Yey compared with WT littermate controls.

### Single-cell RNA sequencing of Dp(10)2Yey hippocampus

A pilot single-cell RNA sequencing (scRNA-seq) experiment was conducted to identify differentially expressed genes (DEGs) and altered gene set pathways in the Dp(10)2Yey model. Fourteen cell clusters were defined, 11 of which were identified by known markers^52^ of microglia, oligodendrocytes, astrocytes, oligodendrocyte precursor cells, endothelial cells, neurons, and ependymal cells (Figure S11A). Differential expression analysis of the scRNA-seq data revealed 2,336 DEGs in the Dp(10)2Yey cells compared with WT controls (Dp(10)2Yey, n = 2; WT, n = 2, adjusted p value <0.01) (Figure S11B). The DEGs were particularly enriched in microglia and neurons; however, the DEGs identified had low log2 fold-changes (<0.5) and therefore were not considered of interest. Gene set enrichment analysis (GSEA) was used to determine whether a defined set of genes showed statistically significant concordant differences between Dp(10)2Yey and WT cells, by focusing on groups of genes that share common biological function or regulation (Subramanian et al., 2005). The widely used Hallmark gene set from the Molecular Signatures Database (MSigDB) was used for further enrichment analysis by GSEA. In total 60 Hallmark pathways were upregulated in the Dp(10)2Yey cells compared with WT cells (FWER <0.05). The enriched pathways include the complement cascade, myogenesis, adipogenesis, tumor necrosis factor-α signaling via nuclear factor (NF)-κB, mTorC1 signaling, and oxidative phosphorylation (Table S3).

## Discussion

Here, we describe cognitive impairments, alterations in neural activity, and the expression of specific interneuron subtypes in Dp(10)2Yey mice, which carry a segmental duplication of Hsa21 orthologous segment on Mmu10 composed of only 37 protein coding genes.^32^ These results extend and complement our previous description of distinct alterations in behavior and oscillatory activity within and between the HPC and mPFC in three different DS mouse models.^16^ In that study, we showed that male Dp(10)2Yey mice do not exhibit spontaneous alternation on the T-maze, consistent with a deficit in hippocampal spatial memory function. Here, we replicate that finding in a different cohort of female Dp(10)2Yey mice, thus demonstrating that both sexes share this behavioral phenotype.

We have now shown that male Dp(10)2Yey mice show reduced mobility in novel open field environments, which could result from increased anxiety-like behavior. As such, it is tempting to speculate that the reduced propensity to spontaneously alternate between goal arms on the T-maze may also reflect anxiety, manifesting as a preference to return to the familiar, previously visited arm rather than the novel, unexplored arm. This finding is reminiscent of our previous behavioral results from Dp1Tyb mice, which showed a reluctance to complete the spontaneous alternation paradigm, despite intact memory function. Further tests are needed to dissociate the relative influence of increased anxiety and apathy on behavior in these animals.

In this study, we showed evidence for females exhibiting stronger anxiety-like behavior. Indeed, an increasing number of studies show sex differences in anxiety and depression behavior in rodents,^53^^,^^54^^,^^55^ in humans,^56^^,^^57^ and even in zebrafish.^58^ Several hypotheses have been proposed to explain the higher vulnerability of females to anxiety.^59^ These include that the circuit involving anxiety behavior (1) is more sensitive in females, (2) is sex specific, and (3) is mediating different behavioral performances. Furthermore, it has been hypothesized that males and females engage different circuits to achieve the same behavioral outcome, known as a convergent sex difference (for summary, see Ref. ^59^). Our findings underscore the need for further work to include both sexes and suggest that sex differences in HPC-mPFC neural activity may underlie behavioral differences relevant to anxiety-like behavior.

To investigate the possible basis of impaired spatial memory function in these animals, we examined the incidence of SWR events in HPC and mPFC during the “baseline” rest period before the first run, and the “consolidation” period between the first and second runs of each trial on the T-maze. To our surprise, we found that the incidence rate of mPFC, but not HPC, SWRs was increased in male Dp(10)2Yey mice during the memory consolidation period. This may appear counterintuitive, as an increased rate of SWRs in the HPC is typically associated with successful memory consolidation. However, the role of SWRs in the mPFC is unclear and aberrant activity could be having a deleterious effect or, alternatively, the increased rate of SWRs could be a compensatory mechanism due to the failure of other processes necessary for spatial memory. Indeed, the relationship between SWRs in the LFP and task-related neural activity remains to be established.

Elsewhere, we found that hippocampal theta-gamma PAC was reduced when Dp(10)2Yey mice explored a novel environment. We previously reported that the same group of animals showed reduced PAC during the spontaneous alternation task.^16^ Again, these results demonstrate that reduced PAC (in particular during stationary periods) in Dp(10)2Yey mice is not chronic but behaviorally dependent—manifesting only in novel environments. Interestingly, both hippocampal theta power and theta-gamma PAC were also correlated with the proportion of time spent mobile in the periphery of a novel environment—but in different directions across Dp(10)2Yey mice and their WT littermates. Although the relationship between hippocampal theta power during movement and the propensity to explore the periphery of an environment is not clear, previous studies have demonstrated a positive correlation between ventral hippocampal theta power and anxiety-like behavior.^28^^,^^60^

In this study, we focused on the innate levels of anxiety in mutant DS mice. Previous studies have described increased theta power in the rodent HPC and mPFC during exposure to anxiogenic environments.^28^^,^^61^ In such environments, animals typically spent a greater fraction of time in the perimeter (i.e., exhibit thigmotaxis). As such, we sought to correlate features of the LFP with the amount of time spent in the periphery of our open field environment, and with the fraction of time in the periphery that the animal remained immobile, another strong behavioral expression of fear or anxiety.

To look for a potential mechanism underlying these electrophysiological differences between Dp(10)2Yey and WT mice, we examined the expression of different classes of interneurons in the HPC and mPFC. By employing a novel technique—the affordable optical clearing and immunolabelling method^62^—we were able to sample 90%–100% of the dorsal HPC and mPFC tissues. This allows us both to retain tissue integrity and to gain more accurate 3D representations of cellular environments using fewer animals. We found a significant loss of CR-expressing interneurons in the HPC. CR-expressing interneurons play a crucial role in the generation of synchronous, rhythmic hippocampal activity by controlling other interneurons terminating on different dendritic and somatic compartments of principal cells.^63^ In addition, these cells regulate the output of the CA1 feedback inhibitory circuitry, controlling the spike timing of oriens-lacunosum-moleculare (OLM) cells to pace their activity at theta frequency.^64^ Their synapses onto OLM cells express α5-GABA(A) receptors, which have been shown to modulate anxiety-like behaviors and spatial memory.^65^ As such, these cells are a strong candidate for establishing and modulating PAC in local hippocampal circuits.

This highlights a potential cellular mechanism for our observations of reduced HPC PAC in novel environments and during spontaneous alternation. Decreased expression of CR interneurons is associated with functional decline of inhibitory activity in the HPC and cognitive deficits.^66^^,^^67^ Interestingly, reduced cell density of CR- (but not PV-) expressing cells has been observed in postmortem superior temporal gyrus of young adult male individuals with DS.^68^ In addition, using DS pluripotent stem cells, DS progenitors differentiated into fewer CR-expressing cells compared with isogenic controls.^68^^,^^69^ These results suggest that altered interneuron expression in DS is modulated specifically by genes expressed in three copies in Dp(10)2Yey mice.

In addition, we observed an increased density of NPY-expressing interneurons in mPFC. These cells have a powerful impact on network dynamics because of their crucial role as an endogenous regulator of neuronal excitability. Therefore, increased expression of NPY-expressing interneurons may account for our observations of abnormal SWR activity in the mPFC of Dp(10)2Yey mice. Collectively, these data suggest that alterations in the expression of specific interneurons in the HPC and mPFC may contribute to the observed phenotypes of cognitive impairment and altered neural activity, thereby demonstrating the potential for an underlying inhibitory contribution in the Dp(10)2Yey mouse model of DS.

Finally, results from a small pilot study using GSEA screening of scRNA-seq from HPC showed that a total of 60 Hallmark pathways were upregulated in the Dp(10)2Yey model compared with WT. Of these pathways, some are involved in the regulation of memory function, such as the complement and mammalian target of rapamycin (mTOR) signaling pathways, whereas others are relevant to DS in a wider context, for example, adipogenesis and myogenesis. The Hallmark complement pathway was upregulated in the cell clusters identified as microglia, astrocytes, oligodendrocytes, and neurons in the Dp(10)2Yey model. Resting microglia communicate with neurons and regulate synaptic pruning through the C1q, C3, and CR3 complement pathways,^70^ and this process plays a critical role in the regulation of learning and memory by mediating neuronal function.^71^^,^^72^ The microglia and oligodendrocyte cell clusters also showed upregulation of the mTOR signaling pathway; mTOR, a serine/threonine protein kinase and component of mTOR complex 1 (mTORC1), plays an important role in memory and synaptic plasticity by regulating nucleotide and protein synthesis.^73^ Several studies have demonstrated that rapamycin, the main mTOR antagonist, improves memory and cognition when administered chronically in mice and rats,^74^^,^^75^^,^^76^ and mTOR is hyperactivated in DS human frontal cortex,^77^ HPC,^78^ and human DS fibroblasts.^79^

Candidate genes within the duplicated region of the Dp(10)2Yey model that may have a role in the upregulation of these key pathways include *S100b*, *Prmt2*, and *Adarb1*. S100B, predominantly produced by astrocytes and microglia, triggers the complement cascade via NF-κB,^80^ and NF-κB-activated astroglial release of complement protein C3 can compromise neuronal morphology and function.^81^ Furthermore, S100B mediates activation of phosphorylated mTOR signaling through the Receptor for Advanced Glycation Endproducts.^82^ Transgenic mice overexpressing human *S100B* from its endogenous promoter display increased repetitive behavior and impaired performance in the Morris water maze, harm avoidance, and social interaction tests.^83^^,^^84^ More importantly, S100B levels are elevated in the brain of people with DS^85^^,^^86^ and in the HPC of the Dp(10)2Yey mouse model (unpublished data). PRMT2 has a role in neuritic arbor formation and dendritic spine induction, maturation, and plasticity,^87^ and human PRMT orthologs play a part in cell proliferation via mTOR signaling.^88^ Similarly, in humans ADARB1 increases cell proliferation and migration by regulating mTOR signaling.^89^ Our preliminary data were generated from a small pilot study (Dp(10)2Yey, n = 2; WT, n = 2), therefore our conclusions are constrained by the low sample number. However, the results identify altered pathways in the Dp(10)2Yey model that are biologically relevant to the dysfunction in working memory phenotype and therefore warrant further investigation.

In summary, we have undertaken behavioral and electrophysiological phenotyping of Dp(10)2Yey mice and identified changes in behavior, neural activity, and interneuron densities in HPC and mPFC that must arise from dosage sensitivity of one or more of the 37 genes in the duplicated region. This extends our understanding of the basis of cognitive dysfunction in DS and lays the groundwork for a final effort to identify the dosage-sensitive gene(s) underlying these phenotypes. The gene(s) identified will not only provide better understanding of cell and molecular mechanisms underlying developmental intellectual disability in DS but also have the potential to translate to important routes for phenotype-modifying therapies.

### Limitations of the study

We showed that female Dp(10)2Yey mice exhibit impaired spatial memory in the spontaneous alternation task; however, unlike male Dp(10)2Yey mice, these mice did not have depth-recording electrodes implanted, and so we could not determine if the electrophysiological properties of female Dp(10)2Yey mice are similar to those in male mice. Indeed, there is a need for further work to investigate possible sex differences in HPC-mPFC neural activity. A pilot study containing a small number of animals was used for the GSEA screening of scRNA-seq, therefore our conclusions are constrained by the low sample number. Nevertheless, the results provide potential targets for altered pathways in the Dp(10)2Yey model that are biologically relevant to the DS phenotype. Last, we have described the association between abnormal histopathology, aberrant neural dynamics, and cognitive impairments, but further work is needed to demonstrate causality, in particular, by using appropriate interventions.

## STAR★Methods

### Key resources table

**Table:** 

REAGENT or RESOURCE	SOURCE	IDENTIFIER
**Antibodies**		

Goat anti-Rabbit 800 nm	LI-COR Biosciences	926-32211; RRID:AB_621843
Goat anti-Rabbit 600 nm	LI-COR Biosciences	926-68071; RRID:AB_10956166
Goat anti-Mouse 800 nm	LI-COR Biosciences	926-32210; RRID:AB_621842
Goat anti-Mouse 600 nm	LI-COR Biosciences	926-68020; RRID:AB_10706161
Rabbit anti-GAPDH	Sigma aldrich	G9545; RRID:AB_796208
Mouse anti-B-actin	Proteintech	60008-1-Ig; RRID:AB_2289225
Mouse anti-Drebrin	Enzo Life Science	ADI-NBA-110-E; RRID:AB_2039073
Rabbit anti-PSD95	Abcam	ab18258; RRID:AB_444362
Rabbit anti-Synapsin1	Cell Signaling	5297; RRID:AB_2616578D
Immunohistology	Muza et al.^62^	N/A

**Chemicals, peptides, and recombinant proteins**		

NuPAGE™ 12% Bis-Tris polyacrylamide gels	Thermo Fisher Scientific	NP0336BOX
NuPAGE™ MOPS SDS Running Buffer	Thermo Fisher Scientific	NP0001
NuPAGE™ MES SDS Running Buffer	Thermo Fisher Scientific	NP0002
Intercept® (PBS) Blocking Buffer	LI-COR Biosciences	927-7000

**Critical commercial assays**		

RNAseq	Adult Brain Dissociation Kit	Miltenyi Biotec Cat# 130-107-677
Trans-Blot Turbo transfer kit	Bio-Rad	170-4270

**Deposited data**		

scRNA sequencing data	This paper	GEO: GSE214273

**Experimental models: Organisms/strains**		

Dp1Tyb (Dp(16Lipi-Zbtb21)1TybEmcf)	This paper	N/A
Dp(10)2Yey (Dp(10Prmt2-Pdxk)1Yey)	This paper	N/A
Dp(17)3Yey (Dp(17Abcg1-Rrp1b)3Yey)	This paper	N/A

**Software and algorithms**		

LWDAQ Software	Open Source Instruments, Brandeis, Boston, USA	http://alignment.hep.brandeis.edu/Software/
Custom MATLAB scripts	This paper	N/A
SPSS 24	Statistical Product and Service Solutions, IBM	https://www.ibm.com/analytics/spss-statistics-software
ESTIMATION STATISTICS	ESTIMATION STATS	https://www.estimationstats.com/#/
Prism	Graphpad	https://www.graphpad.com/scientific-software/prism/
Custom R scripts	This paper	N/A
Custom Python scripts	This paper	N/A
Seurat	Butler et al.^90^	(v3.1.0)
scCATCH	Shao et al.^91^	(v2.1)
Image Studio Lite^TM^ Software	LI-COR Biosciences	(v5.2.5)

### Resource availability

#### Lead contact

Further information and requests should be directed to and will be fulfilled by lead contact: Pishan Chang (pi-shan.chang@bristol.ac.uk), and corresponding author: Matthew Walker (m.walker@ucl.ac.uk) or Elizabeth Fisher (elizabeth.fisher@ucl.ac.uk).

#### Materials availability

This study did not generate new unique reagents.

### Experimental model and subject details

Dp(10)2Yey mice^32^^,^^92^ were maintained within a facility at University College London and Cardiff University. Animals were maintained in colonies as hemizygous mutants backcrossed for over ten generations to C57BL/6J, with age-matched WT littermates used as controls. All experiments were undertaken using male aged 3–4 month old (behavior and electrophysiology) and female animals aged 3–4 month old (behavior only), blinded to genotype, which was decoded after experimental analysis and reconfirmed using an independent DNA sample isolated from a postmortem tail.

All experiments were performed in accordance with the United Kingdom Animal (Scientific Procedures) Act 1986. All experiments conform to the relevant regulatory standards is included in this STAR Methods section. Reporting is based on the ARRIVE 2 Guidelines for Reporting Animal Research developed by the National Center for Replacement, Refinement and Reduction of Animals in Research, London, United Kingdom. Mice were housed in controlled conditions in accordance with guidance issued by the Medical Research Council on “Responsibility in the use of animals in bioscience research“ (2019) and all experiments were carried out under Licence from the UK Home Office and with Local Ethical Review Board (AWERB) approval. Mice were housed in individually ventilated cages (IVC) of 2–5 age-matched animals under controlled environmental conditions (24–25°C; 50%–60% humidity; 12 h light/dark cycle) with free access to food and water.

### Method details

#### Surgical preparation and transmitter implantation for long-term recording

Mice were anesthetized with 2.5%–3% isoflurane (Abbot, AbbVie Ltd., Maidenhead, UK) in 100% oxygen (flow rate of 1–1.5 L/min) via gas anesthesia mask (Model 906, David Kopf Instruments, CA, USA) from a recently calibrated vaporizer (Harvard Apparatus, Cambridge, MA). Body temperature was maintained with a heat blanket during surgery. A transmitter (A3028A, Open Source Instruments, Brandeis, Boston, USA)^93^ was implanted subcutaneously with the depth recording electrodes (a 125 μm diameter teflon-insulated stainless steel electrode with 10kOhm impedance, Open Source Instruments, Brandeis, Boston, USA) positioned in mPFC (1.8 mm anterior, 0.4 mm lateral, 1.5 mm ventral) and dorsal HPC (1.85 mm posterior, 1.25 mm lateral, 1.45 mm ventral; Paxinos, 2012). The reference electrode was implanted over the cerebellum posterior to lambda. The whole assembly was held in place with dental cement (Simplex Rapid, Acrylic Denture Polymer, UK). At the end of the experiment, recording electrode locations were verified, and LFP data were only included in the study if electrode tips were located in mPFC and dorsal HPC. In total, LFP data from just one animal was excluded because the recording site was outside the target region. Due to the relatively large diameter of the recording electrode, and prolonged recording period, it is difficult to specify the precise region of dorsal HPC from which recordings were made; it is likely that our measurements reflect field potentials summated over a relatively large region. Nonetheless, we estimate that ∼57% of recordings were made from CA1 stratum radiatum, ∼9% from CA1 stratum oriens, and ∼34% from dentate gyrus. A subcutaneous injection of bupivacaine and metacam was provided for post-surgical pain management. At the end of surgery, enrofloxacin (5 mg/kg, Baytril, Bayer health care) and pre-warm saline (0.5–1 mL) were administered subcutaneously. The animals were placed in a temperature-controlled (25°C) recovery chamber until ambulatory and closely monitored at least 1–2 h before returning to their home cage to allow recovery for at least 14 days after surgery. The transmitter, which has no adverse effects,^94^ was implanted for data recordings. During all recording sessions, continuous LFP recordings were recorded (bandpass filter: 0.2 Hz–160 Hz, 512Hz sampling rate with 16-bit resolution) using LWDAQ Software (Open Source Instruments, Brandeis, Boston, USA). Animals were carefully monitored daily and were euthanized at the end of the experiment with pentobarbital (25 mg/kg).

#### Behavioral tests

Behavioural tests were performed in the T-maze and the open field. The interval between tests was 2 days. The apparatus were custom made. The behavior analysis was performed using Any-Maze and custom MATLAB scripts. Animals were transferred to the testing room for 1–2 h before each experiment to habituate to the environment and achieve an optimal state of arousal.

#### Behavioral testing: T-maze spontaneous alternation

Working memory function in male mice from each strain and associated age-matched WT controls was assessed using the spontaneous alternation paradigm in an enclosed T-maze apparatus.^95^ This test is suitable for working memory and route-learning capabilities. The protocol has been described in detail in.^16^ In brief, the T-maze is an enclosed apparatus in the form of a T placed horizontally. Animals are placed at the base of the T and allowed to choose one of the goal arms abutting the other end of the stem. If two runs are given in quick succession, on the second run, the rodent is expected to choose the arm not visited before, reflecting the memory of the first choice.

#### Behavioral testing: Open field

The open field test is widely used to test exploratory behavior and general activity of mice and rats.^96^ This test was carried out in an arena (20 cm wide × 40 cm length x 20 cm height) divided into 2 sections with an interval of 45 min. In the first section (novel environment), the mice were placed in the arena and allowed to explore it for 5 min. The test was used to evaluate spontaneous locomotor activity and anxiety (time spent moving in the central area and in the corner areas).^97^^,^^98^ In the second session, mice were placed in the same test area (familiar environment), and were allowed to explore freely for 5 min.

#### EEG pre-processing

First, we used the Generalized Extreme Studentised Deviate test to identify outlier samples (i.e. artifacts) in the EEG signal, we replaced artifacts of ≤5 samples duration by linear interpolation, we marked any remaining artifacts so that the corresponding EEG data was excluded from all subsequent analyses. Finally, we applied a 1Hz high pass filter (using a 400th order FIR filter) to the resultant signal, and z-scored the amplitude of that signal to ensure consistency across recordings.

Next, for open field sessions, we extracted tracking data from video recordings (sampled at 25Hz) to estimate the location and movement speed of the animal in each time bin. We then split the data into movement (defined as ≥1s periods of continuous ≥5 cm/s movement) and stationary (defined as ≥1s periods of continuous <5 cm/s movement) epochs for subsequent analysis.

#### Generating power spectra

First, we detrended the signal from each of the movement or stationary epochs identified above, and generated power spectra using the fast Fourier transform. The resultant spectra were then smoothed with a 2Hz Gaussian kernel, averaged across epochs in each condition (i.e. across all movement or stationary periods), and peak power and frequency in the 6–12Hz theta band was extracted from the average spectra.

#### Estimating phase-amplitude coupling

For illustrative purposes, cross-coherence images were computed as described previously (Chang et al., 2020): first, we used a five cycle Morlet wavelet to generate power time series for each frequency band for each of the movement or stationary epochs described above. Next, we computed coherence between the raw EEG signal and each power time series. Finally, we averaged cross-coherence images across epochs in each condition (i.e. across all movement or stationary periods).

Phase-amplitude coupling between specific pairs of phase and amplitude frequency bands was computed by band-pass filtering the EEG signal in each frequency band (using a 400th order FIR filter), using the Hilbert transform to extract the amplitude of the high frequency signal and the phase of the low frequency signal, respectively, and then computing the mean amplitude of the high frequency in each of twenty evenly spaced phase bins of the low frequency signal. The resultant vector length of this distribution was subsequently computed for each epoch, and then averaged across epochs in each condition (i.e. across all movement or stationary periods).

#### Sharp-wave ripple detection

Sharp-wave ripple (SWR) events were detected by first band-pass filtering the pre-processed EEG signal in the >150Hz range (using a 400th order FIR filter), then using the Hilbert transform to extract amplitude at each time point, smoothing the amplitude time series with a 15ms Gaussian filter, and z-scoring the resultant signal. Candidate SWR events were subsequently defined as ≥40ms periods with amplitude Z ≥ 0 and peak amplitude Z ≥ 3. Any candidate events that were separated by ≤ 40ms were merged, and any candidate events with duration ≥500ms were discarded.

#### Immunohistochemistry

##### Brain collection

Animals were anesthetized with 100% isoflurane via inhalation and thereafter transcardially perfused with 0.01 M PBS followed by 4% ice-cold formalin in PBS, prior to extracting whole brain and immerse fixation in 4% formalin for 24 h at 4°C. The tissue was then transferred to PBS +0.05% sodium azide solution for long term storage at 4°C. Tissue was sectioned using a Vibratome (Leica VT1000S) at 1 mm thickness.

#### Optical clearing and immunolabelling brain slices

Optical clearing and immunolabelling brain slices were performed using the ABSOC method, as described in Muza et al.,.^62^ In separate experiments, calretinin (CR), parvalbumin (PV), and neuropeptide-Y positive cells were immunolabelled using rabbit anti-calretinin (Abcam – ab244299, 1:5000), rabbit anti-parvalbumin (Abcam – ab11427, 1:5000), and rabbit anti-neuropeptide-Y (Abcam – ab10980, 1:5000) antibodies, respectively. All primary antibodies were probed with goat anti-rabbit Alexa Fluor 633 (ThermoFisher – A21071, 1:1000) secondary antibodies.

#### Confocal microscopy

Following optical clearing and immunolabelling, brain slices were mounted on glass microscopic slides fitted inside an 800 μm silicone spacer (Sylgard – 01673921) with BABB solution (33% v/v benzyl alcohol (Sigma-Aldrich – 305197) and 66% v/v benzyl benzoate (Sigma-Aldrich – B6630)). Brain slices were then imaged with on a Leica SP8 confocal microscope with an x10, 0.4NA air objective. Two images per brain slice were acquired – the brain slice autofluorescence detected at wavelength 488 nm, and the cell signal detected at wavelength 633 nm.

#### Image post-processing

Raw images were stitched using Leica SP8 Navigator tool and adjusted using Fiji (ImageJ 1.51 v9).

#### Image registration and cell density quantification

Custom-made ImageJ, python, and R scripts were developed to assign segmented cells to their appropriate brain region. This script registers the brain slice autofluorescence data to the Allen Mouse Brain Common Coordinate Framework (CCFv3 –^99^) to annotate brain regions, individually segments cells, and combines cell coordinates and anatomical regions to produce a table describing cell density for a given region in a brain slice.

#### Golgi-Cox staining

A modified Golgi-Cox method^100^ was used to analyze dendritic spine density. After being removed from the skulls, brains were incubated in Golgi-Cox solution (1% potassium dichromate, 1% mercury chloride, 0.8% potassium chromate) for 48 h at room temperature in the dark. This solution was then renewed and tissue was immersed for 3 more weeks. Thereafter, brains were washed with distilled water (dH_2_0) and maintained in 90° ethanol for 30 min before being cut coronally (thickness of 200 μm) in 70% ethanol using a vibratome. Brain slices were washed with dH_2_0, reduced in 16% ammonia for 1 h and fixed in 1% sodium thiosulfate for 7 min. After another wash with dH_2_0, they were placed on microscope slides, dehydrated (50%, 70%, 80% and 100% ethanol (3 min each), 2x isopropanol: ethanol (2:1; 5 min each), isopropanol (5 min) and 2x xylene (5 min each)) and mounted with Omnimount (National Diagnostics).

Dendritic spine density was determined in the secondary apical dendrites of hippocampal CA1 pyramidal cells. Selected neurons were captured using Axio Observer 7 (Zeiss) at a resolution of 15.5077 pixels per micron. For each mouse (n = 4–5 per group), 3 dendrites of 9 different neurons were used for the analysis.

#### Protein extracts

Total protein extracts were obtained from mouse HPC. Hippocampal tissue was homogenized in lysis buffer containing 2% SDS, 10 mM Tris-HCl (pH7.5), phosphatase inhibitors (1 mM NaF and 0.1 mM Na_3_VO_4_) and the commercial Complete Protease Inhibitor Cocktail (Roche) using a T 10 basic ULTRA-TURRAX® (Ika). Homogenates were sonicated for 2 min, left 20 min on ice, vortexed and centrifuged for 13 min at 15,700 g and 8°C. Supernatants were kept and maintained at −80°C until use. Protein concentration was determined using the Pierce BCA Protein Assay kit (Thermo Fisher Scientific).

#### Immunoblotting

Total hippocampal extracts were used to analyze the levels of targeted proteins by immunoblotting. They were mixed with 4X NuPAGE™ LDS Sample Buffer (Thermo Fisher Scientific) containing β-mercaptoethanol, denaturalized at 95°C for 5 min and resolved onto NuPAGE™ 4 to 12% Bis-Tris polyacrylamide gels (Thermo Fisher Scientific) using NuPAGE™ MOPS SDS Running Buffer (20X; Thermofisher) for medium-size to big targeted-proteins and NuPAGE™ MES SDS Running Buffer (20X; Thermofisher) for small ones. Proteins were then transferred onto 0.2 μm nitrocellulose membranes using Trans-Blot Turbo transfer system (Bio-Rad) at 1.3 A for 10 min. Membranes were blocked for 1 h with Intercept® (PBS) Blocking Buffer and incubated overnight at 4°C with the corresponding primary antibodies (rabbit anti-Synapsin1 (Cell Signaling; 1:60000), rabbit anti-PSD95 (abcam, 1:1000), mouse anti-Drebrin (Enzo Life Sciences, 1:1000), rabbit anti-GAPDH (Sigma Aldrich, 1:200000), beta-actin (Proteintech, 1:300000) diluted in blocking buffer. After blocking, membranes were washed 3 times (10 min each) in PBST (0.05% Tween in PBS) and incubated for 1 h with the pertinent IRDye-secondary antibody (LI-COR Biosciences) diluted in blocking buffer. Finally, membranes were washed twice with PBST and once with PBS for 10 min. Antibody binding was visualised by Odyssey® CLx Imaging System (LI-COR Biosciences) and Image Studio LiteTM Software (LI-COR Biosciences, version 5.2.5) was used for protein quantification.

#### Single-cell RNA sequencing

##### Sample preparation

Single-cell preparation was carried out on 2 consecutive days, at the same time on each day, with each experiment comprising 1 male Dp(10)2Yey and 1 male wild-type (WT) littermate at 3 months old. Mice were killed by cervical dislocation; their brains were removed and the hippocampi microdissected in ice-cold Dulbecco’s PBS (D-PBS). The dissected hippocampi were dissociated using the Adult Brain Dissociation Kit (Miltenyi Biotec #130-107-677) according to the manufacturer’s instructions, with the following exceptions: the enzyme mixes were added sequentially and a gentleMACS^TM^ Octo Dissociator, followed by incubation in a 37°C orbital shaker, was used for the mechanical dissociation steps. Tissue was dissociated in pre-warmed Enzyme Mix 1 (E1) on the gentleMACS Octo Dissociator for 36 s, then transferred to a 37 °C orbital shaker and incubated for 15 min at 100 rpm. Enzyme Mix 2 (E2) was added to each sample and tissue was dissociated on the gentleMACS^TM^ Octo Dissociator for 30 s, followed by a 10 min incubation in a 37 °C orbital shaker at 100 rpm. Samples were then dissociated on the gentleMACS^TM^ Octo Dissociator for 59 s, followed by a final 10 min incubation in a 37 °C orbital shaker at 100 rpm. Cell suspensions were applied to 70 μm pre-moistened cell strainers and cells were collected by centrifugation for 10 min at 300 xg, 4 °C. Cell pellets were resuspended in ice-cold D-PBS using wide-bore, pre-moistened tips, prior to debris removal, which was carried out according to the manufacturer’s instructions. Final cell pellets were resuspended in 0.04% BSA in D-PBS (Miltenyi Biotec MACS BSA #130-091-376). Cell viability was assessed using a NanoEntek Eve Automated Cell Counter. The single-cell suspension was then loaded into the 10x Chromium.

##### Sequencing and mapping

One library for each of the 4 samples was prepared according to the manufacturer’s instructions (single cell 3′ v1 protocol, 10x Genomics). The 10x Genomics Cell Ranger software (v.3.0.2)^101^ was used to de-multiplex Illumina BCL output, create FASTQ files and generate single cell feature counts for each library using a custom mouse genome (mm10–3.0.0) as reference. All subsequent analyses were performed in R programming language (v.3.6.1) using the Seurat (v.3.1.0) package.^90^ Genes were removed if they were expressed in 3 or less cells and cells with less than 200 genes detected were also removed. Data was integrated following Seurat’s Canonical Correlation Analysis approach (CCA). For each sample the top 2000 most variable genes were selected for data integration using CCA. 50 dimensions were used for dimensional reduction using t-distributed stochastic neighbor embedding (t-SNE) and cluster calling. Clusters were visualized using the Uni-form Mani-fold Approximation and Projection (UMAP). Upon initial examination two clusters with high expression levels of the hemoglobin alpha-1 (Hba-a1) gene were identified as red blood cells, these were removed. This was done before normalization and integration and the analysis was repeated with the same parameters using a resolution of 0.2 to define clusters. Cluster markers were identified using the function "FindAllMarkers" with default parameters, and cluster identity was established by manual curation of literature together with scCATCH.^91^

##### Differential expression and gene set enrichment analysis

In order to find genes differentially expressed between WT and Dp(10)2Yey samples within each cluster, the DESeq2 implementation in Seurat "FindMarkers" function was used. Gene set enrichment analysis (GSEA) was carried out using Cluster Profiler,^102^^,^^103^ using a minimum gene set size of 15, maximum gene set size of 500,000 and a cut off of p.adjust < 0.05, with the following Gene Collections "c2.cp.v7.2.symbols.gmt", "c5.go.bp.v7.2.symbols.gmt" and "h.all.v7.2.symbols.gmt" downloaded from the Broad Institute.

### Quantification and statistical analysis

Detailed statistical analysis was performed using GraphPad Prism 6 (GraphPad Software), SPSS (Statistical Product and Service Solutions, IBM), and R-Studio (R version 3.6.3). All data are presented as mean ± SEM. Comparisons of means were performed using one way ANOVA with Tukey post hoc test or two-way ANOVA with Holm post hoc test were appropriate, if the data were normally distributed; Kruskal-Wallis test with post hoc Dunn’s multiple comparisons test if the data were not normally distributed (with the Shapiro-Wilk test used to assess normality of the data distributions).

Detailed statistical analysis was performed using Estimation statistics (open source estimation program available on https://www.estimationstats.com).^104^ Estimation statistics report mean differences (effect size) with expressions of uncertainty (confidence interval estimates). In this method, each paired mean difference is plotted as a bootstrap sampling distribution, using 5000 bootstrap samples and the confidence intervals are bias corrected and accelerated. The p value(s) reported is the likelihood(s) of observing the effect size(s), if the null hypothesis of zero difference is true. For each permutation p value, 5000 reshuffles of the control and test labels were performed; p < 0.05 is considered a significant difference. Pearson correlation and linear regression were applied to calculate the behavior correlation with electrophysiological data. The significance threshold for all correlation tests was set at p < 0.05.

## Data Availability

• scRNA sequencing data have been deposited at GEO and are publicly available as of the date of publication. Accession numbers are listed in the key resources table. • This paper does not report original code. • Any additional information required to reanalyse the data reported in this paper is available from the lead contact upon request.

## Supplemental information

Document S1. Figures S1–S11 and Table S1–S3

Data S1. Detailed statistics, related to Figures 1–6, S2–S7, and S10

## References

[1] de Graaf G., Buckley F., Skotko B.G. (2015). Estimates of the live births, natural losses, and elective terminations with Down syndrome in the United States. Am. J. Med. Genet..

[2] Hanney M., Prasher V., Williams N., Jones E.L., Aarsland D., Corbett A., Lawrence D., Yu L.-M., Tyrer S., Francis P.T. (2012). Memantine for dementia in adults older than 40 years with Down’s syndrome (MEADOWS): a randomised, double-blind, placebo-controlled trial. Lancet.

[3] Wu J., Morris J.K. (2013). Trends in maternal age distribution and the live birth prevalence of Down’s syndrome in England and Wales: 1938–2010. Eur. J. Hum. Genet..

[4] Loane M., Morris J.K., Addor M.-C., Arriola L., Budd J., Doray B., Garne E., Gatt M., Haeusler M., Khoshnood B. (2012). Twenty-year trends in the prevalence of Down syndrome and other trisomies in Europe: impact of maternal age and prenatal screening. Eur. J. Hum. Genet..

[5] Grieco J., Pulsifer M., Seligsohn K., Skotko B., Schwartz A. (2015). Down syndrome: cognitive and behavioral functioning across the lifespan. Am. J. Med. Genet. C Semin. Med. Genet..

[6] Lott I.T., Dierssen M. (2010). Cognitive deficits and associated neurological complications in individuals with Down’s syndrome. Lancet Neurol..

[7] Dykens E.M., Shah B., Davis B., Baker C., Fife T., Fitzpatrick J. (2015). Psychiatric disorders in adolescents and young adults with Down syndrome and other intellectual disabilities. J. Neurodev. Disord..

[8] Foley K.R., Bourke J., Einfeld S.L., Tonge B.J., Jacoby P., Leonard H. (2015). Patterns of depressive symptoms and social relating behaviors differ over time from other behavioral domains for young people with down syndrome. Medicine (Baltim.).

[9] Rosso M., Fremion E., Santoro S.L., Oreskovic N.M., Chitnis T., Skotko B.G., Santoro J.D. (2020). Down syndrome disintegrative disorder: a clinical regression syndrome of increasing importance. Pediatrics.

[10] Vicari S., Pontillo M., Armando M. (2013). Neurodevelopmental and psychiatric issues in Down’s syndrome: assessment and intervention. Psychiatr. Genet..

[11] Smith K., Hogan A.L., Will E., Roberts J.E. (2021). Attention bias and prodromal anxiety symptoms in toddlers with fragile X syndrome and down syndrome. Am. J. Intellect. Dev. Disabil..

[12] Gupta M., Dhanasekaran A.R., Gardiner K.J. (2016). Mouse models of Down syndrome: gene content and consequences. Mamm. Genome.

[13] Herault Y., Delabar J.M., Fisher E.M.C., Tybulewicz V.L.J., Yu E., Brault V. (2017). Rodent models in Down syndrome research: impact and future opportunities. Dis. Model. Mech..

[14] Stagni F., Giacomini A., Guidi S., Ciani E., Bartesaghi R. (2015). Timing of therapies for Down syndrome: the sooner, the better. Front. Behav. Neurosci..

[15] Lana-Elola E., Watson-Scales S., Slender A., Gibbins D., Martineau A., Douglas C., Mohun T., Fisher E.M., Tybulewicz V.L. (2016). Genetic dissection of Down syndrome-associated congenital heart defects using a new mouse mapping panel. Elife.

[16] Chang P., Bush D., Schorge S., Good M., Canonica T., Shing N., Noy S., Wiseman F.K., Burgess N., Tybulewicz V.L.J. (2020). Altered hippocampal-prefrontal neural dynamics in mouse models of down syndrome. Cell Rep..

[17] Jacinto L.R., Cerqueira J.J., Sousa N. (2016). Patterns of theta activity in limbic anxiety circuit preceding exploratory behavior in approach-avoidance conflict. Front. Behav. Neurosci..

[18] Alemany-González M., Gener T., Nebot P., Vilademunt M., Dierssen M., Puig M.V. (2020). Prefrontal–hippocampal functional connectivity encodes recognition memory and is impaired in intellectual disability. Proc. Natl. Acad. Sci. USA..

[19] Ruggiero R.N., Rossignoli M.T., Marques D.B., de Sousa B.M., Romcy-Pereira R.N., Lopes-Aguiar C., Leite J.P. (2021). Neuromodulation of hippocampal-prefrontal cortical synaptic plasticity and functional connectivity: implications for neuropsychiatric disorders. Front. Cell. Neurosci..

[20] Wirt R.A., Hyman J.M. (2017). Integrating spatial working memory and remote memory: interactions between the medial prefrontal cortex and hippocampus. Brain Sci..

[21] Benchenane K., Tiesinga P.H., Battaglia F.P. (2011). Oscillations in the prefrontal cortex: a gateway to memory and attention. Curr. Opin. Neurobiol..

[22] Guitart-Masip M., Barnes G.R., Horner A., Bauer M., Dolan R.J., Duzel E. (2013). Synchronization of medial temporal lobe and prefrontal rhythms in human decision making. J. Neurosci..

[23] Jones M.W., Wilson M.A. (2005). Theta rhythms coordinate hippocampal–prefrontal interactions in a spatial memory task. PLoS Biol..

[24] Siapas A.G., Lubenov E.V., Wilson M.A. (2005). Prefrontal phase locking to hippocampal theta oscillations. Neuron.

[25] Young C.K., McNaughton N. (2009). Coupling of theta oscillations between anterior and posterior midline cortex and with the hippocampus in freely behaving rats. Cereb. Cortex.

[26] Floresco S.B., Seamans J.K., Phillips A.G. (1997). Selective roles for hippocampal, prefrontal cortical, and ventral striatal circuits in radial-arm maze tasks with or without a delay. J. Neurosci..

[27] Wang G.-W., Cai J.-X. (2008). Reversible disconnection of the hippocampal-prelimbic cortical circuit impairs spatial learning but not passive avoidance learning in rats. Neurobiol. Learn. Mem..

[28] Adhikari A., Topiwala M.A., Gordon J.A. (2010). Synchronized activity between the ventral hippocampus and the medial prefrontal cortex during anxiety. Neuron.

[29] Jacinto L.R., Reis J.S., Dias N.S., Cerqueira J.J., Correia J.H., Sousa N. (2013). Stress affects theta activity in limbic networks and impairs novelty-induced exploration and familiarization. Front. Behav. Neurosci..

[30] Seidenbecher T., Laxmi T.R., Stork O., Pape H.-C. (2003). Amygdalar and hippocampal theta rhythm synchronization during fear memory retrieval. Science.

[31] Lesting J., Narayanan R.T., Kluge C., Sangha S., Seidenbecher T., Pape H.-C. (2011). Patterns of coupled theta activity in amygdala-hippocampal-prefrontal cortical circuits during fear extinction. PLoS One.

[32] Yu T., Li Z., Jia Z., Clapcote S.J., Liu C., Li S., Asrar S., Pao A., Chen R., Fan N. (2010). A mouse model of Down syndrome trisomic for all human chromosome 21 syntenic regions. Hum. Mol. Genet..

[33] Horsch M., Seeburg P.H., Adler T., Aguilar-Pimentel J.A., Becker L., Calzada-Wack J., Garrett L., Götz A., Hans W., Higuchi M. (2011). Requirement of the RNA-editing enzyme ADAR2 for normal physiology in mice ∗. J. Biol. Chem..

[34] Hou W., Nemitz S., Schopper S., Nielsen M.L., Kessels M.M., Qualmann B. (2018). Arginine methylation by PRMT2 controls the functions of the actin nucleator cobl. Dev. Cell.

[35] Joensuu T., Tegelberg S., Reinmaa E., Segerstråle M., Hakala P., Pehkonen H., Korpi E.R., Tyynelä J., Taira T., Hovatta I. (2014). Gene expression alterations in the cerebellum and granule neurons of Cstb−/− mouse are associated with early synaptic changes and inflammation. PLoS One.

[36] Xie Y.-F., Belrose J.C., Lei G., Tymianski M., Mori Y., MacDonald J.F., Jackson M.F. (2011). Dependence of NMDA/GSK-3β mediated metaplasticity on TRPM2 channels at hippocampal CA3-CA1 synapses. Mol. Brain.

[37] Battaglia F.P., Benchenane K., Sirota A., Pennartz C.M.A., Wiener S.I. (2011). The hippocampus: hub of brain network communication for memory. Trends Cogn. Sci..

[38] Jadhav S.P., Rothschild G., Roumis D.K., Frank L.M. (2016). Coordinated excitation and inhibition of prefrontal ensembles during awake hippocampal sharp-wave ripple events. Neuron.

[39] Tang W., Shin J.D., Frank L.M., Jadhav S.P. (2017). Hippocampal-prefrontal reactivation during learning is stronger in awake compared with sleep states. J. Neurosci..

[40] Tort A.B.L., Kramer M.A., Thorn C., Gibson D.J., Kubota Y., Graybiel A.M., Kopell N.J. (2008). Dynamic cross-frequency couplings of local field potential oscillations in rat striatum and hippocampus during performance of a T-maze task. Proc. Natl. Acad. Sci. USA..

[41] Cabral H.O., Vinck M., Fouquet C., Pennartz C.M.A., Rondi-Reig L., Battaglia F.P. (2014). Oscillatory dynamics and place field maps reflect hippocampal ensemble processing of sequence and place memory under NMDA receptor control. Neuron.

[42] Klausberger T., Somogyi P. (2008). Neuronal diversity and temporal dynamics: the unity of hippocampal circuit operations. Science.

[43] Lisman J.E., Jensen O. (2013). The theta-gamma neural code. Neuron.

[44] Mann E.O., Paulsen O. (2007). Role of GABAergic inhibition in hippocampal network oscillations. Trends Neurosci..

[45] Hentschke H., Benkwitz C., Banks M.I., Perkins M.G., Homanics G.E., Pearce R.A. (2009). Altered GABAA,slow inhibition and network oscillations in mice lacking the GABAA receptor beta3 subunit. J. Neurophysiol..

[46] Han S., Tai C., Jones C.J., Scheuer T., Catterall W.A. (2014). Enhancement of inhibitory neurotransmission by GABAA receptors having α2,3-subunits ameliorates behavioral deficits in a mouse model of autism. Neuron.

[47] Lewis D.A., Curley A.A., Glausier J.R., Volk D.W. (2012). Cortical parvalbumin interneurons and cognitive dysfunction in schizophrenia. Trends Neurosci..

[48] Luscher B., Fuchs T., Rudolph U. (2015). In Advances in Pharmacology.

[49] Benavides-Piccione R., Ballesteros-Yáñez I., de Lagrán M.M., Elston G., Estivill X., Fillat C., DeFelipe J., Dierssen M. (2004). On dendrites in Down syndrome and DS murine models: a spiny way to learn. Prog. Neurobiol..

[50] Coyle J.T., Oster-Granite M.L., Gearhart J.D. (1986). The neurobiologie consequences of down syndrome. Brain Res. Bull..

[51] Marin-Padilla M. (1972). Structural abnormalities of the cerebral cortex in human chromosomal aberrations: a Golgi study. Brain Res..

[52] Zeisel A., Muñoz-Manchado A.B., Codeluppi S., Lönnerberg P., La Manno G., Juréus A., Marques S., Munguba H., He L., Betsholtz C. (2015). Cell types in the mouse cortex and hippocampus revealed by single-cell RNA-seq. Science.

[53] De Oliveira Sergio T., Wetherill L., Kwok C., Khoyloo F., Hopf F.W. (2021). Sex differences in specific aspects of two animal tests of anxiety-like behavior. Psychopharmacology (Berl.).

[54] Scholl J.L., Afzal A., Fox L.C., Watt M.J., Forster G.L. (2019). Sex differences in anxiety-like behaviors in rats. Physiol. Behav..

[55] Lee S.E., Greenough E.K., Oancea P., Fonken L.K., Gaudet A.D. (2022). Anxiety-like behaviors in mice unmasked: revealing sex differences in anxiety using a novel light-heat conflict test. bioRxiv.

[56] Burani K., Nelson B.D. (2020). Gender differences in anxiety: the mediating role of sensitivity to unpredictable threat. Int. J. Psychophysiol..

[57] Kelly M.M., Tyrka A.R., Anderson G.M., Price L.H., Carpenter L.L. (2008). Sex differences in emotional and physiological responses to the trier social stress test. J. Behav. Ther. Exp. Psychiatry.

[58] Genario R., Giacomini A.C.V.V., de Abreu M.S., Marcon L., Demin K.A., Kalueff A.V. (2020). Sex differences in adult zebrafish anxiolytic-like responses to diazepam and melatonin. Neurosci. Lett..

[59] Bangasser D.A., Cuarenta A. (2021). Sex differences in anxiety and depression: circuits and mechanisms. Nat. Rev. Neurosci..

[60] Schoenfeld T.J., Kloth A.D., Hsueh B., Runkle M.B., Kane G.A., Wang S.S.-H., Gould E. (2014). Gap junctions in the ventral hippocampal-medial prefrontal pathway are involved in anxiety regulation. J. Neurosci..

[61] Wells C.E., Amos D.P., Jeewajee A., Douchamps V., Rodgers J., O’Keefe J., Burgess N., Lever C. (2013). Novelty and anxiolytic drugs dissociate two components of hippocampal theta in behaving rats. J. Neurosci..

[62] Muza P.M., Perez-Gonzalez M., Noy S., Kurosawa M., Katsouri L., Tybulewicz V.L.J., Fisher E.M., West S.J. (2022). Affordable optical clearing and immunolabelling in mouse brain slices. Res. Sq..

[63] Gulyás A.I., Hájos N., Freund T.F. (1996). Interneurons containing calretinin are specialized to control other interneurons in the rat Hippocampus. J. Neurosci..

[64] Tyan L., Chamberland S., Magnin E., Camiré O., Francavilla R., David L.S., Deisseroth K., Topolnik L. (2014). Dendritic inhibition provided by interneuron-specific cells controls the firing rate and timing of the hippocampal feedback inhibitory circuitry. J. Neurosci..

[65] Magnin E., Francavilla R., Amalyan S., Gervais E., David L.S., Luo X., Topolnik L. (2019). Input-specific synaptic location and function of the α5 GABA_A_ receptor subunit in the mouse CA1 hippocampal neurons. J. Neurosci..

[66] Baglietto-Vargas D., Moreno-Gonzalez I., Sanchez-Varo R., Jimenez S., Trujillo-Estrada L., Sanchez-Mejias E., Torres M., Romero-Acebal M., Ruano D., Vizuete M. (2010). Calretinin interneurons are early targets of extracellular amyloid-β pathology in PS1/AβPP alzheimer mice Hippocampus. J. Alzheimers Dis..

[67] Giesers N.K., Wirths O. (2020). Loss of hippocampal calretinin and parvalbumin interneurons in the 5XFAD mouse model of alzheimer’s disease. ASN Neuro.

[68] Giffin-Rao Y., Sheng J., Strand B., Xu K., Huang L., Medo M., Risgaard K.A., Dantinne S., Mohan S., Keshan A. (2022). Altered patterning of trisomy 21 interneuron progenitors. Stem Cell Rep..

[69] Huo H.-Q., Qu Z.-Y., Yuan F., Ma L., Yao L., Xu M., Hu Y., Ji J., Bhattacharyya A., Zhang S.-C., Liu Y. (2018). Modeling down syndrome with patient iPSCs reveals cellular and migration deficits of GABAergic neurons. Stem Cell Rep..

[70] Cornell J., Salinas S., Huang H.-Y., Zhou M. (2022). Microglia regulation of synaptic plasticity and learning and memory. Neural Regen. Res..

[71] Hong S., Beja-Glasser V.F., Nfonoyim B.M., Frouin A., Li S., Ramakrishnan S., Merry K.M., Shi Q., Rosenthal A., Barres B.A. (2016). Complement and microglia mediate early synapse loss in Alzheimer mouse models. Science.

[72] Wang C., Yue H., Hu Z., Shen Y., Ma J., Li J., Wang X.-D., Wang L., Sun B., Shi P. (2020). Microglia mediate forgetting via complement-dependent synaptic elimination. Science.

[73] Laplante M., Sabatini D.M. (2009). mTOR signaling at a glance. J. Cell Sci..

[74] Fu J., Wang H., Gao J., Yu M., Wang R., Yang Z., Zhang T. (2017). Rapamycin effectively impedes melamine-induced impairments of cognition and synaptic plasticity in wistar rats. Mol. Neurobiol..

[75] Halloran J., Hussong S.A., Burbank R., Podlutskaya N., Fischer K.E., Sloane L.B., Austad S.N., Strong R., Richardson A., Hart M.J., Galvan V. (2012). Chronic inhibition of mammalian target of rapamycin by rapamycin modulates cognitive and non-cognitive components of behavior throughout lifespan in mice. Neuroscience.

[76] Sabran-Cohen T., Bright U., Mizrachi Zer-Aviv T., Akirav I. (2021). Rapamycin prevents the long-term impairing effects of adolescence Δ-9-tetrahydrocannabinol on memory and plasticity in male rats. Eur. J. Neurosci..

[77] Perluigi M., Pupo G., Tramutola A., Cini C., Coccia R., Barone E., Head E., Butterfield D.A., Di Domenico F. (2014). Neuropathological role of PI3K/Akt/mTOR axis in Down syndrome brain. Biochim. Biophys. Acta.

[78] Iyer A.M., van Scheppingen J., Milenkovic I., Anink J.J., Adle-Biassette H., Kovacs G.G., Aronica E. (2014). mTOR hyperactivation in down syndrome Hippocampus appears early during development. J. Neuropathol. Exp. Neurol..

[79] Bordi M., Darji S., Sato Y., Mellén M., Berg M.J., Kumar A., Jiang Y., Nixon R.A. (2019). mTOR hyperactivation in Down Syndrome underlies deficits in autophagy induction, autophagosome formation, and mitophagy. Cell Death Dis..

[80] Reinehr S., Reinhard J., Gandej M., Gottschalk I., Stute G., Faissner A., Dick H.B., Joachim S.C. (2018). S100B immunization triggers NFκB and complement activation in an autoimmune glaucoma model. Sci. Rep..

[81] Lian H., Yang L., Cole A., Sun L., Chiang A.C.-A., Fowler S.W., Shim D.J., Rodriguez-Rivera J., Taglialatela G., Jankowsky J.L. (2015). Nfκb-activated astroglial release of complement C3 compromises neuronal morphology and function associated with alzheimer’s disease. Neuron.

[82] Seguella L., Capuano R., Pesce M., Annunziata G., Pesce M., de Conno B., Sarnelli G., Aurino L., Esposito G. (2019). S100B protein stimulates proliferation and angiogenic mediators release through RAGE/pAkt/mTOR pathway in human colon adenocarcinoma caco-2 cells. Int. J. Mol. Sci..

[83] Whitaker-Azmitia P.M., Wingate M., Borella A., Gerlai R., Roder J., Azmitia E.C. (1997). Transgenic mice overexpressing the neurotrophic factor S-100β show neuronal cytoskeletal and behavioral signs of altered aging processes: implications for Alzheimer’s disease and Down’s syndrome. Brain Res..

[84] Busciglio J., Capone G., O'Bryan J., Gardiner K.J. (2013). Down syndrome: genes, model systems, and progress towards pharmacotherapies and clinical trials for cognitive deficits. Cytogenet. Genome Res..

[85] Netto C.B.O., Portela L.V., Ferreira C.T., Kieling C., Matte U., Felix T., da Silveira T.R., Souza D.O., Gonçalves C.A., Giugliani R. (2005). Ontogenetic changes in serum S100B in Down syndrome patients. Clin. Biochem..

[86] Mito T., Becker L.E. (1993). Developmental changes of S-100 protein and glial fibrillary acidic protein in the brain in down syndrome. Exp. Neurol..

[87] Qualmann B., Kessels M.M. (2021). The role of protein arginine methylation as post-translational modification on actin cytoskeletal components in neuronal structure and function. Cells.

[88] Hwang J.W., Cho Y., Bae G.-U., Kim S.-N., Kim Y.K. (2021). Protein arginine methyltransferases: promising targets for cancer therapy. Exp. Mol. Med..

[89] Behroozi J., Shahbazi S., Bakhtiarizadeh M.R., Mahmoodzadeh H. (2020). ADAR expression and copy number variation in patients with advanced gastric cancer. BMC Gastroenterol..

[90] Butler A., Hoffman P., Smibert P., Papalexi E., Satija R. (2018). Integrating single-cell transcriptomic data across different conditions, technologies, and species. Nat. Biotechnol..

[91] Shao X., Liao J., Lu X., Xue R., Ai N., Fan X. (2020). scCATCH: automatic annotation on cell types of clusters from single-cell RNA sequencing data. iScience.

[92] Yu T., Liu C., Belichenko P., Clapcote S.J., Li S., Pao A., Kleschevnikov A., Bechard A.R., Asrar S., Chen R. (2010). Effects of individual segmental trisomies of human chromosome 21 syntenic regions on hippocampal long-term potentiation and cognitive behaviors in mice. Brain Res..

[93] Chang P., Hashemi K.S., Walker M.C. (2011). A novel telemetry system for recording EEG in small animals. J. Neurosci. Methods.

[94] Chang P., Fabrizi L., Olhede S., Fitzgerald M. (2016). The development of nociceptive network activity in the somatosensory cortex of freely moving rat pups. Cereb. Cortex.

[95] Deacon R.M.J. (2006). Housing, husbandry and handling of rodents for behavioral experiments. Nat. Protoc..

[96] Crusio W.E. (2001). Genetic dissection of mouse exploratory behaviour. Behav. Brain Res..

[97] Carola V., D’Olimpio F., Brunamonti E., Mangia F., Renzi P. (2002). Evaluation of the elevated plus-maze and open-field tests for the assessment of anxiety-related behaviour in inbred mice. Behav. Brain Res..

[98] Scheich B., Gaszner B., Kormos V., László K., Ádori C., Borbély É., Hajna Z., Tékus V., Bölcskei K., Ábrahám I. (2016). Somatostatin receptor subtype 4 activation is involved in anxiety and depression-like behavior in mouse models. Neuropharmacology.

[99] Wang Q., Ding S.-L., Li Y., Royall J., Feng D., Lesnar P., Graddis N., Naeemi M., Facer B., Ho A. (2020). The allen mouse brain common coordinate framework: a 3D reference atlas. Cell.

[100] Glaser E.M., Van der Loos H. (1981). Analysis of thick brain sections by obverse—reverse computer microscopy: application of a new, high clarity Golgi—nissl stain. J. Neurosci. Methods.

[101] Zheng G.X.Y., Terry J.M., Belgrader P., Ryvkin P., Bent Z.W., Wilson R., Ziraldo S.B., Wheeler T.D., McDermott G.P., Zhu J. (2017). Massively parallel digital transcriptional profiling of single cells. Nat. Commun..

[102] Wu T., Hu E., Xu S., Chen M., Guo P., Dai Z., Feng T., Zhou L., Tang W., Zhan L. (2021). clusterProfiler 4.0: a universal enrichment tool for interpreting omics data. Innovation.

[103] Yu G., Wang L.-G., Han Y., He Q.-Y. (2012). clusterProfiler: an R package for comparing biological themes among gene clusters. OMICS A J. Integr. Biol..

[104] Ho J., Tumkaya T., Aryal S., Choi H., Claridge-Chang A. (2019). Moving beyond P values: data analysis with estimation graphics. Nat. Methods.

