# Supplementary material for: Cognitive impairments in a Down syndrome model with abnormal hippocampal and prefrontal dynamics and cytoarchitecture
Source: iScience. 2023 Jan 28;26(2):106073. doi: 10.1016/j.isci.2023.106073 (PMC9929862; doi:10.1016/j.isci.2023.106073)
Supplement: Document S1. Figures S1–S11 and Table S1–S3 [file mmc1.pdf]

iScience, Volume *26*

Supplemental information

Cognitive impairments in a Down syndrome model with abnormal hippocampal and prefrontal dynamics and cytoarchitecture

Phillip M. Muza, Daniel Bush, Marta Pérez-González, Ines Zouhair, Karen Cleverley, Miriam L. Sopena, Rifdat Aoidi, Steven J. West, Mark Good, Victor L.J. Tybulewicz, Matthew C. Walker, Elizabeth M.C. Fisher, and Pishan Chang

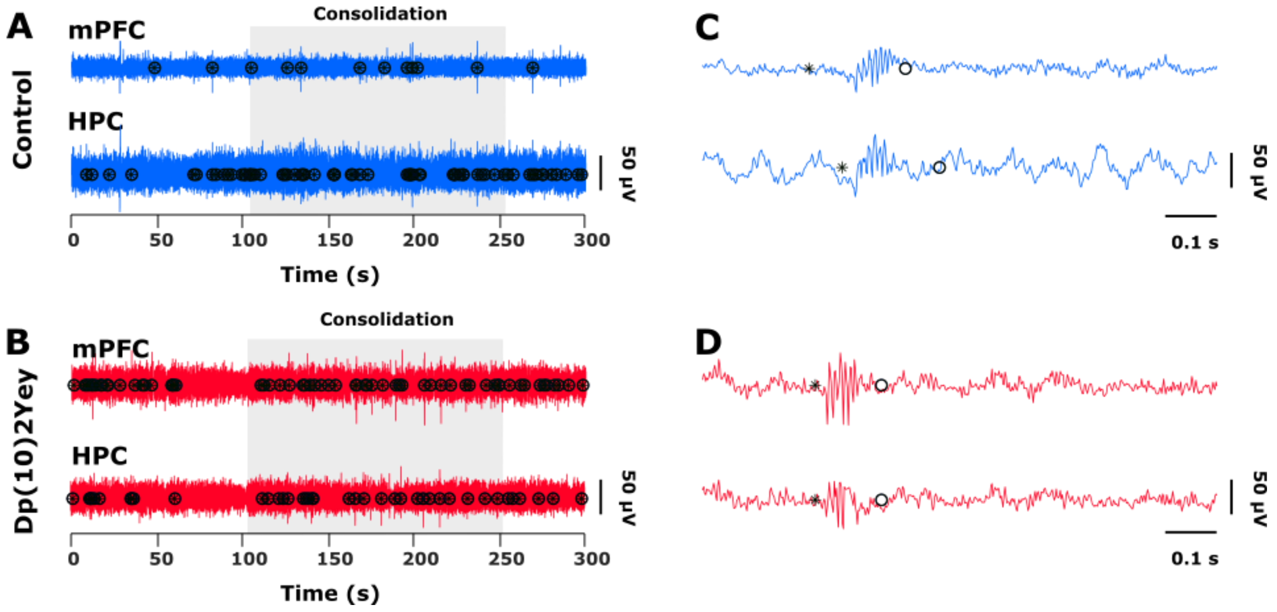

Figure S1: Representative traces of local field potential (LFP). **Related to figure 1**. LFP recorded from mPFC and HPC of wild-type littermates **(A)** and Dp(10)2Yey mice **(B)** during the spontaneous alternation task. The detected SWRs are marked with the symbol ***** (start) +**o** (end). The (C) and (D) show example SWRs at greater time resolution.

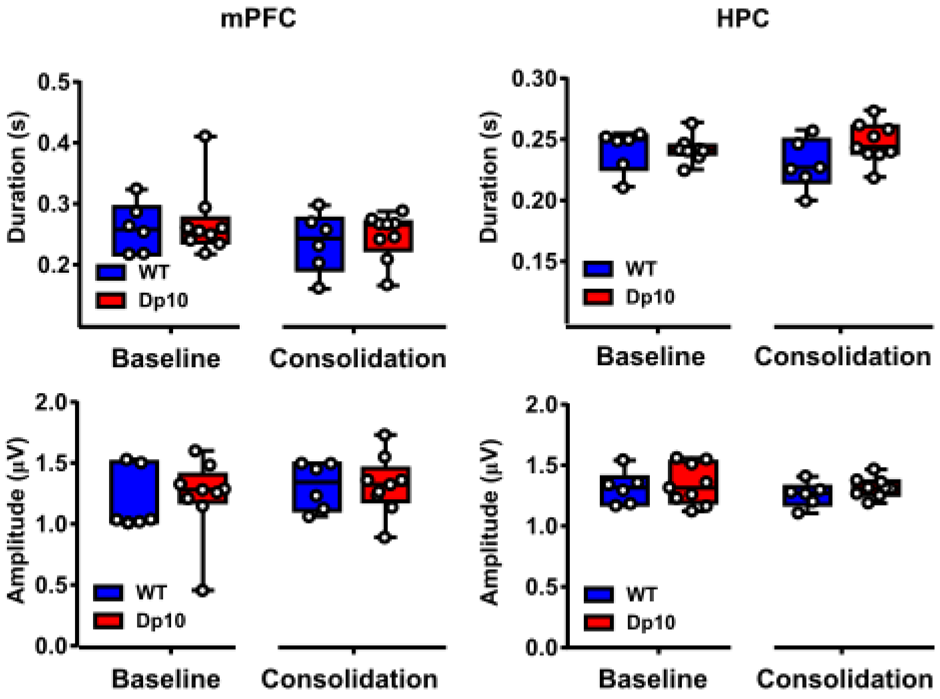

Figure S2: Comparison of sharp-wave ripple (SWR) activity in the hippocampus and mPFC between male Dp(10)2Yey mice (**Dp10**, n=9) and wild-type littermates (**WT**, n=6) during the spontaneous alternation task. **Related to figure 1**. Data are presented as box-whisker plots indicating the median, 25-75th percentiles, and minimum-maximum values with data for individual mice superimposed. Statistical comparisons are assessed using a one-way ANOVA with Tukey's multiple comparison test. All statistical details are presented in Data S1.

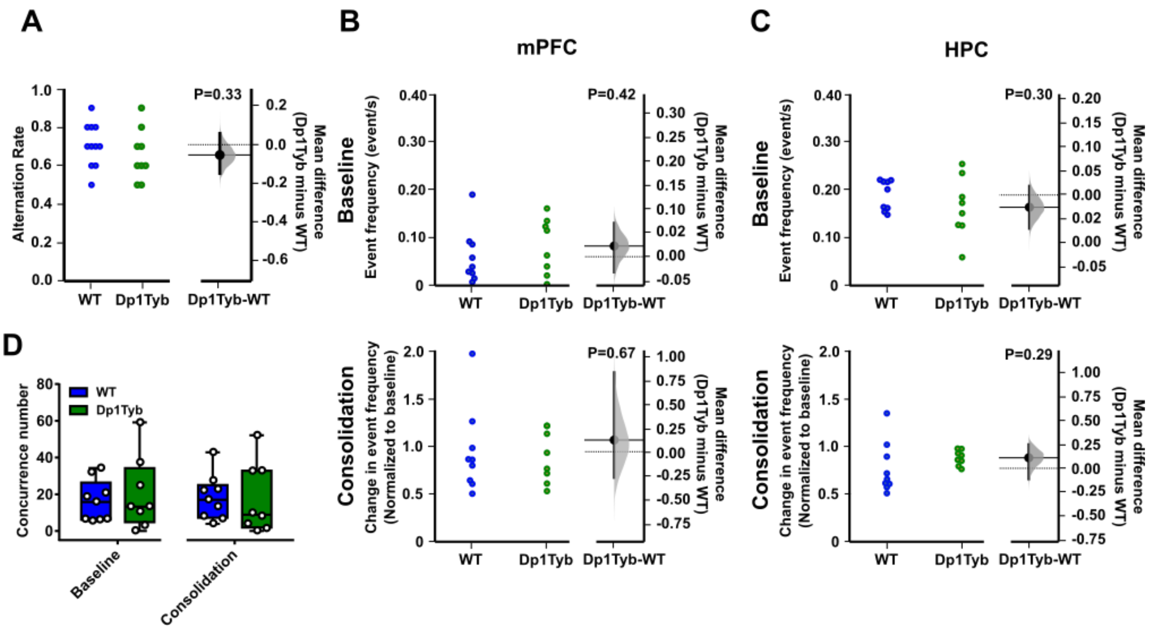

Figure S3: Comparison of sharp-wave ripples (SWR) activity in the hippocampus (HPC) and mPFC between male DP1Tyb mice (**Dp1Tyb**) and wild-type littermates (**WT**) during the spontaneous alternation task. **Related to figure 1. (A)** Behavioural alternation rates are significantly higher than chance level (0.5) in both WT (n=11, W=2.83, p=0.005) and Dp1Tyb (n=9, W=2.38, p=0.017) animals. **(B, C)** Comparison of SWR event incidence rates in mPFC and HPC during baseline and consolidation periods between WT (n=9) and Dp1Tyb (n=8) mice. **(D)** Concurrence of SWR activity between mPFC and HPC. **(A-C)** show a scatter plot of raw data from individual animals in the left-hand panel, and the distribution of paired mean differences in the Cumming estimation plot on the right. In this panel, mean differences are indicated with a large black dot, and 95% confidence intervals are indicated by the ends of the vertical error bars. Statistical analysis was performed using a permutation t-test (with 5000 shuffles). **(D)** shows box-whisker plots indicating the median, 25-75th percentiles, and minimum-maximum values with data for individual mice superimposed. All statistical details are presented in Data S1.

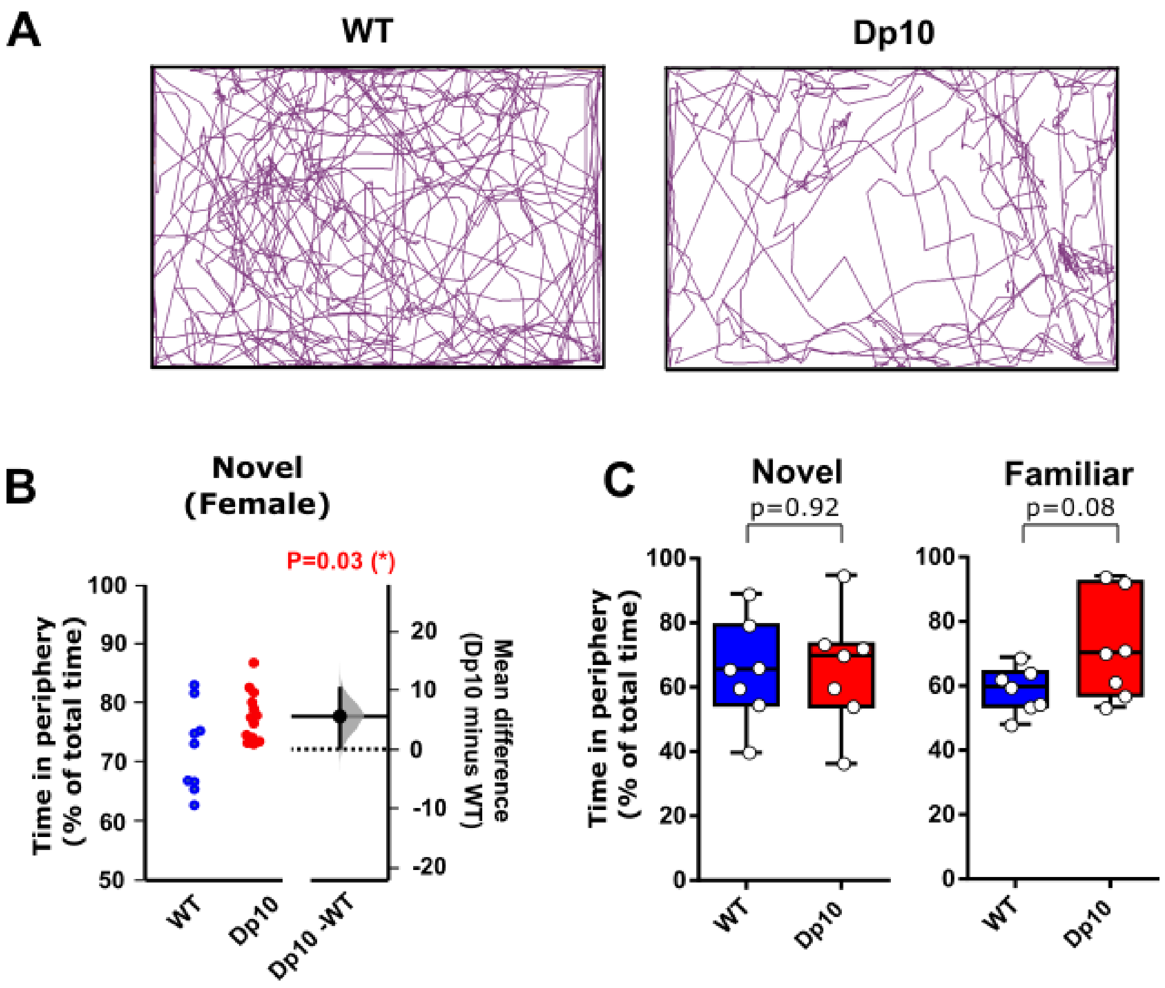

Figure S4: Comparison of behaviour between Dp(10)2Yey (**Dp10**) and wild-type littermates (**WT**) during exploration of an open field environment. **Related to figure 2. (A)** Representative movement paths in a novel open field environment. (**B**) Female Dp(10)2Yey DS mice (n=13) show anxiety-like behaviour in the novel open field environment, manifesting as more time spent in the periphery of the environment than female WT mice (n=9). **(C)** Conversely, male Dp(10)2Yey DS mice (n=7) do not spend more time in the periphery of either novel or familiar environments than male WT mice (n=7). For **(B)**, the mean difference between groups is shown in the accompanying Gardner-Altman estimation plot. Both groups are plotted on the left axis; the mean difference is plotted on a floating axis on the right as a bootstrap sampling distribution. The mean difference is depicted as a dot; the 95% confidence interval is indicated by the limits of the vertical error bar. Statistical analysis was performed using a permutation t-test (with 5000 shuffles). For **(C)**, the data are presented as box-whisker plots indicating the median, 25-75th percentiles, and minimum-maximum values with data for individual mice superimposed. Statistical comparisons are assessed using a one-way ANOVA with Tukey's multiple comparison test. All statistical details are presented in Data S1.

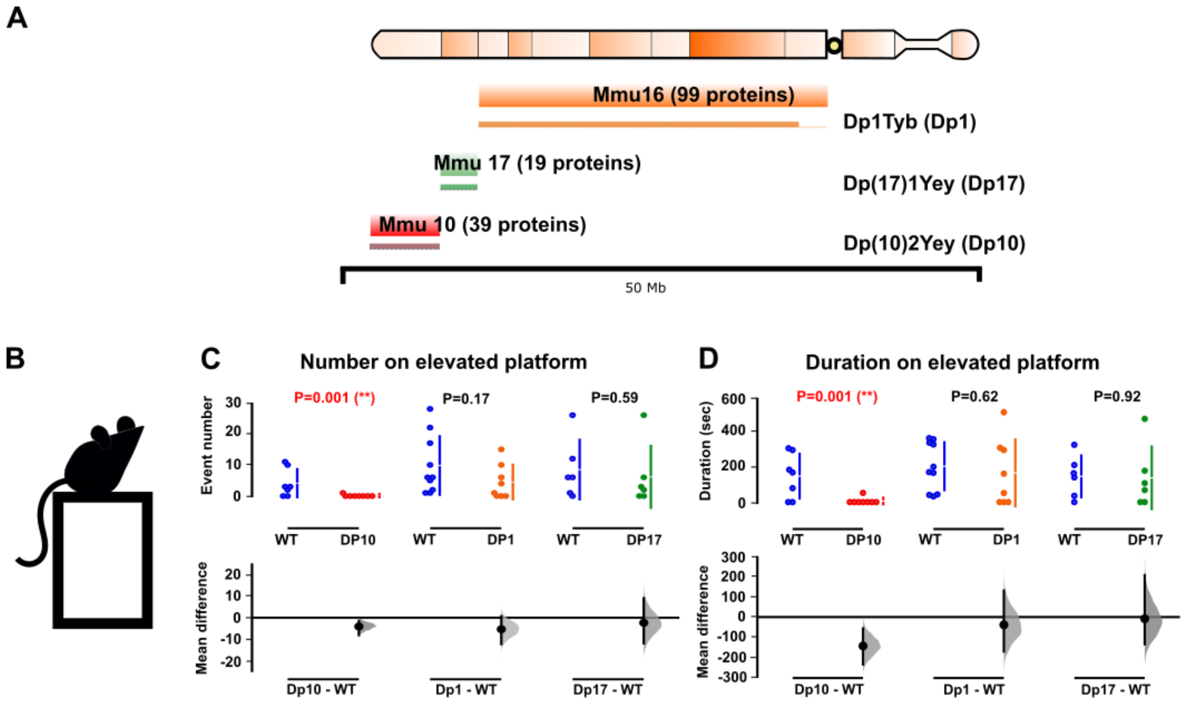

Figure S5: Comparison of behaviour between Dp(10)2Yey (**Dp10**, n=8), Dp1Tyb (**Dp1**, n=8), Dp(17)3Yey (**Dp17**, n=6) and wild-type littermates (**WT**; n=7, n=10 and n=6, respectively) during the elevated-platform test. **Related to figure 2. (A)** Schematic illustration of the different mouse models of Down syndrome used in this test. **(B)** Schematic diagram of the elevated platform task. (**C)** Number of climbing bouts onto the elevated platform for each group. This shows that Dp(10)2Yey mice undertook fewer climb events to the elevated platform compared to WT. **(D)** Duration of time spent on the elevated platform by each group. This shows that Dp(10)2Yey mice spent less time on the elevated platform compared to WT. The paired mean differences for comparison are shown in the Cumming estimation plots below. Each paired mean difference is plotted as a bootstrap sampling distribution. Mean differences are depicted as dots; 95% confidence intervals are indicated by the limits of the vertical error bars. Statistical analysis was performed using the permutation t-test (with 5000 shuffles). All statistical details are presented in Data S1.

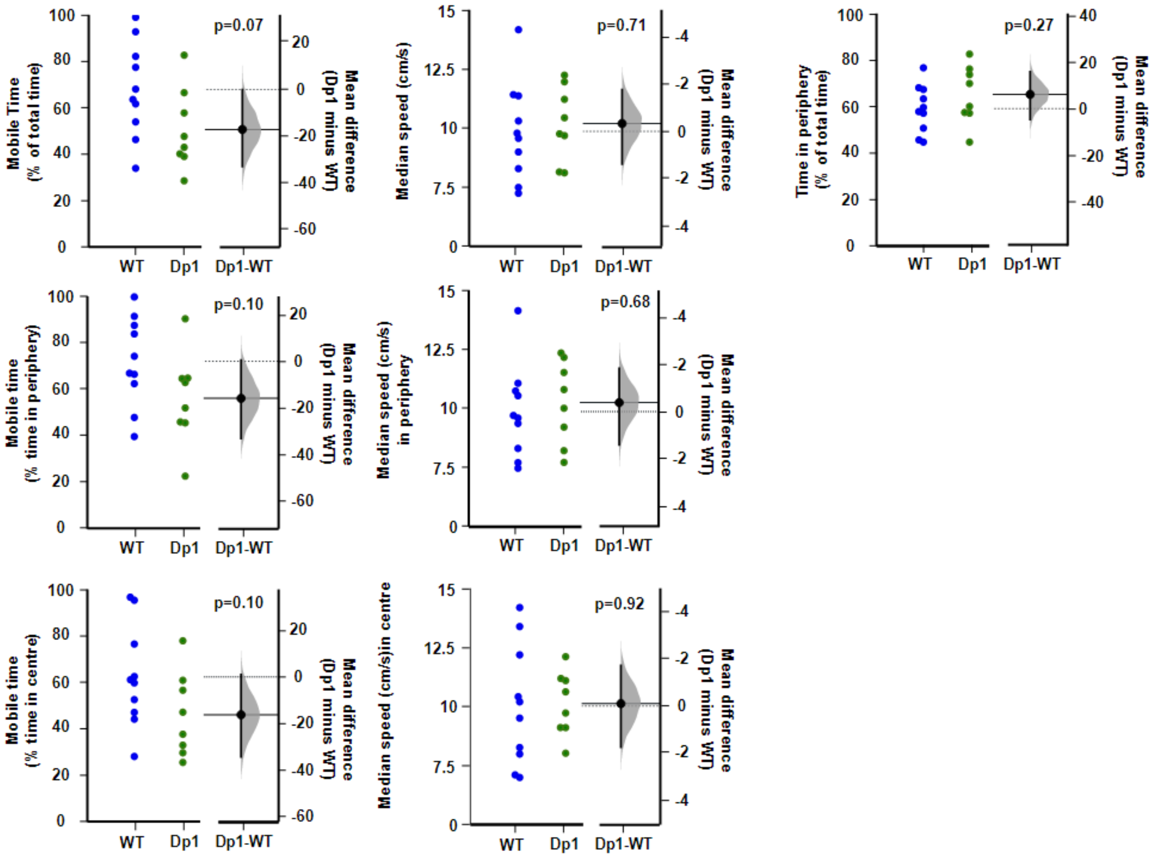

Figure S6: Comparison of behaviour between Dp(1)Tyb (**Dp1, n=8**) and wild-type littermates (**WT, n=10**) during exploration of a novel environment. **Related to figure 2.** Left panel shows a scatter plot of raw data from individual animals; right panel shows the bootstrap sampling distribution of paired mean differences in Gardner-Altman estimation plots. The mean differences are depicted as black dots and the black line shows the 95% confidence interval. Statistical analysis was performed using a permutation test (with 5000 shuffles). All statistical details are presented in Data S1.

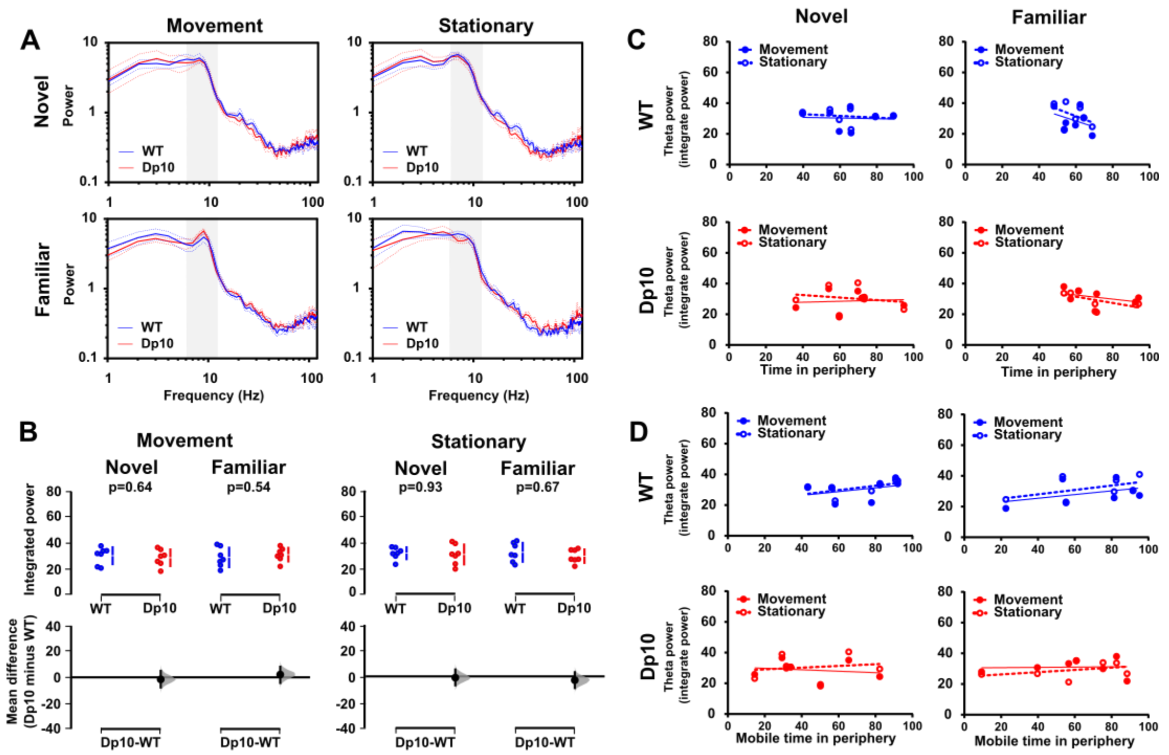

Figure S7: Comparison of mPFC theta power between male Dp(10)2Yey (**Dp10**, n=7) and wild-type littermates (WT, n=7) during exploration of an open field environment. **Related to figure 3.** **(A)** mPFC power spectra for novel and familiar open field environments. Data are presented as mean ± SEM. **(B)** Comparison of integrated theta power during movement and stationary periods between Dp10 and WT mice in novel and familiar open field environments. The paired mean differences for comparisons are shown in the Cumming estimation plots below. Each paired mean difference is plotted as a bootstrap sampling distribution. Mean differences are depicted as dots; 95% confidence intervals are indicated by the limits of the vertical error bars. Statistical analysis was performed using the permutation t-test (with 5000 shuffles). **(C)** Relationship between integrated theta power and total percentage of time spent in the periphery. Continuous line shows the linear regression. **(D)** Relationship between integrated theta power and percentage of time spent mobile while in the periphery. Continuous line shows the linear regression. Pearson correlation coefficients (R) and significance (p values) for **(C, D)** are presented in Supplementary Table 1. All statistical details are presented in Data S1.

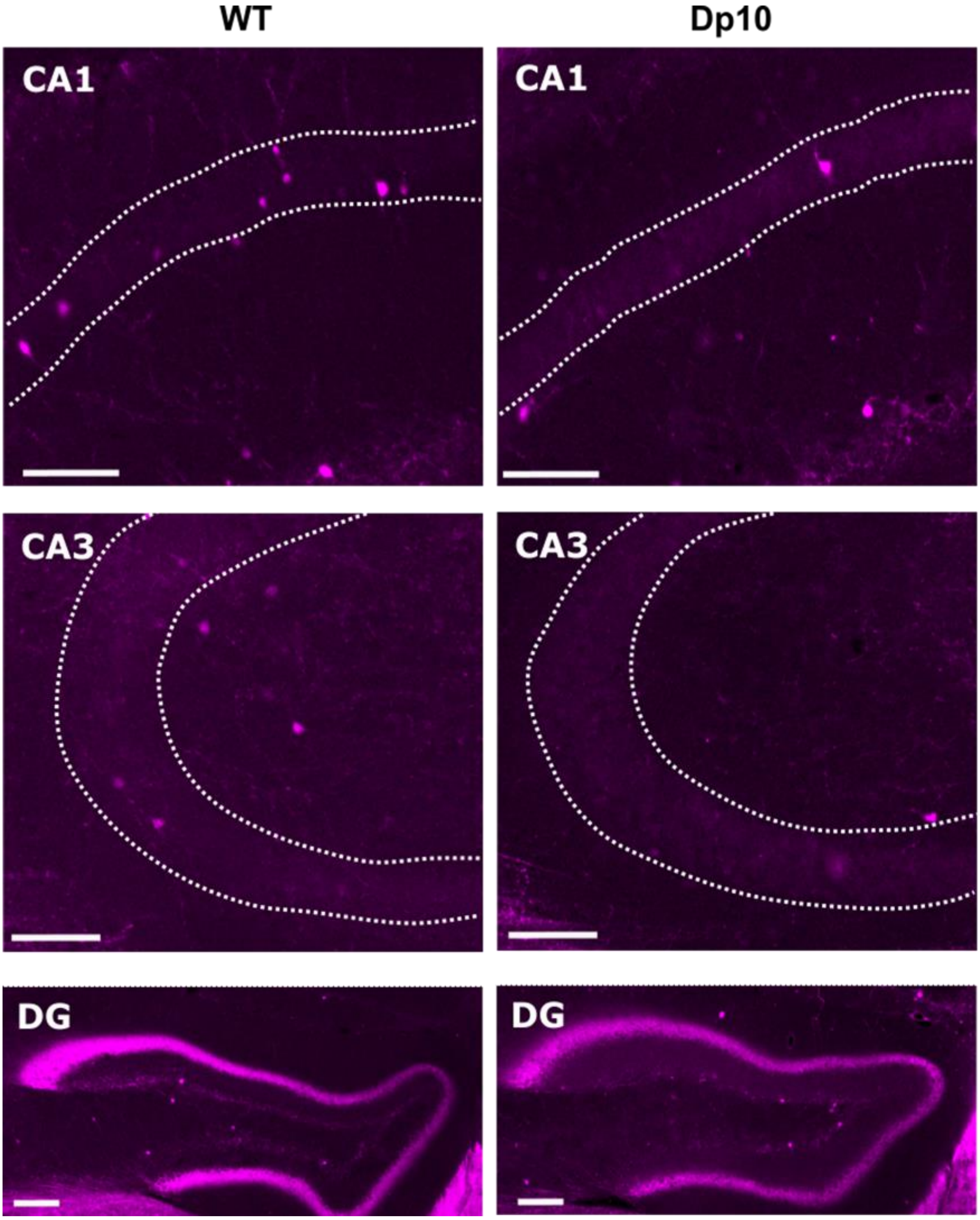

Figure S8: Example of Immunohistochemical staining of CR- expressing interneurons in hippocampal areas CA1, CA3, and DG. **Related to figure 5.** Scare bar indicates 100 µm.

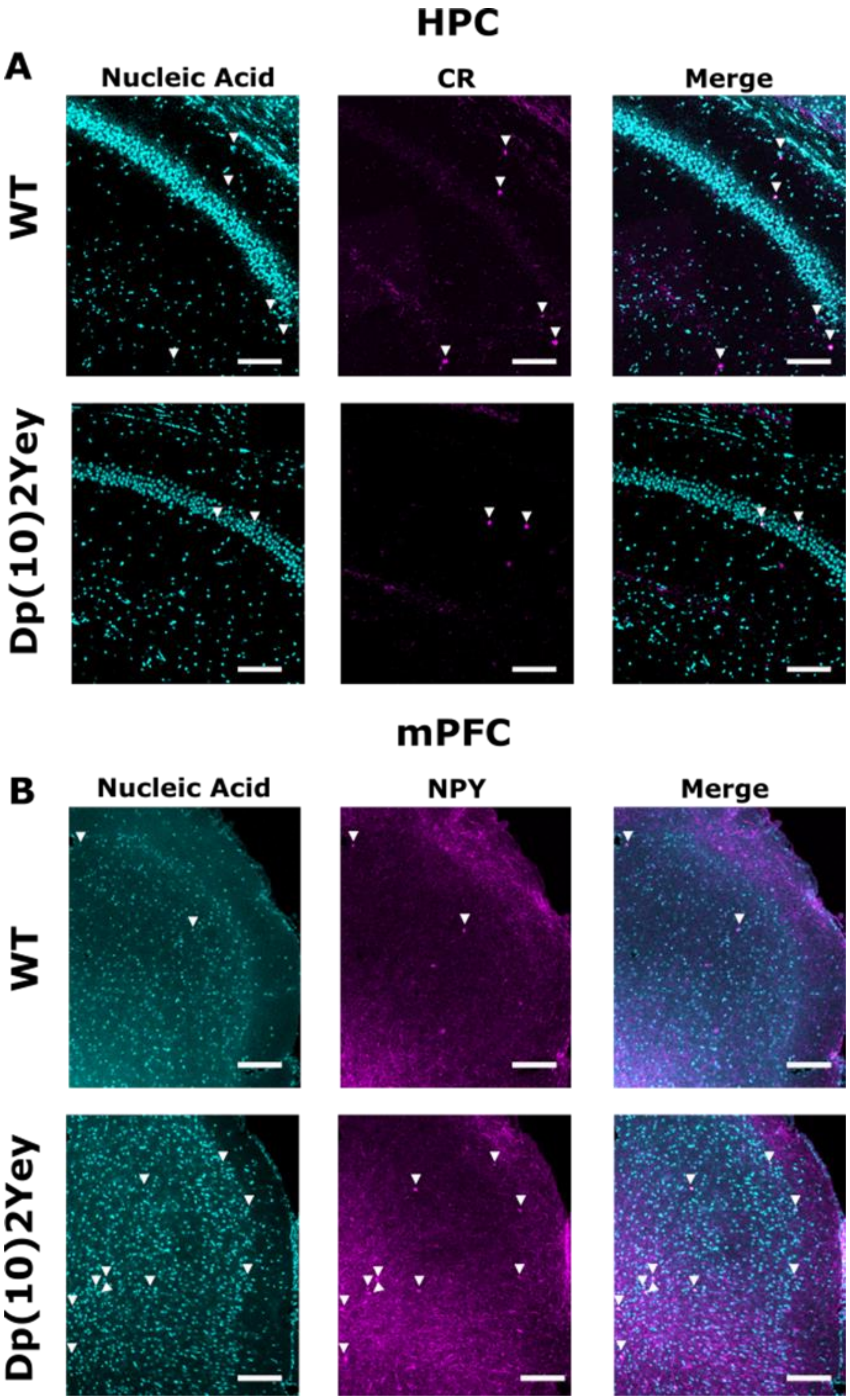

Figure S9: Example of immunofluorescence images of calretinin (CR)-expressing interneurons in hippocampal areas CA1, and Neuropeptide Y (NPY)- expressing interneurons in mPFC. **Related to figure 5.** SYTOX dye were used to stain nuclei acid in fixed tissue material. White arrowheads indicate overlap of cell marker and nuclear stain in the **(A)** HPC CA1 area and **(B)** mPFC area. Scare bar indicates 100 µm. These 2D images were taken using a 10x dry microscope Objective Lens.

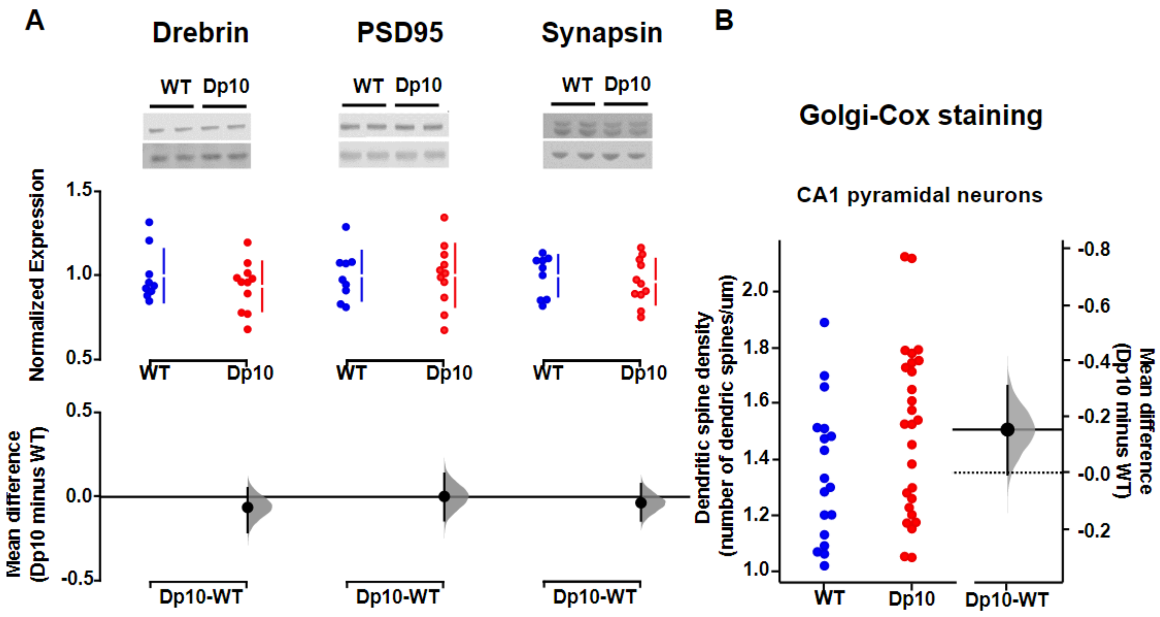

Figure S10: Comparison of dendritic spine morphology and basal synaptic plasticity between male Dp(10)2Yey DS mice (**Dp10**) and their wild-type littermates (**WT**). **Related to figure 5. (A)** The synaptic markers Drebrin, Synapsin, and PSD95 were used to quantify the expression of synaptic connections in Dp(10)2Yey (n=11) and WT (n=9) mice. The paired mean differences for comparisons are shown in the Cumming estimation plots below. Each paired mean difference is plotted as a bootstrap sampling distribution. Mean differences are depicted as dots; 95% confidence intervals are indicated by the limits of the vertical error bars. Statistical analysis was performed using the permutation t-test (with 5000 shuffles). **(B)** Golgi-Cox staining was used to visualize and compare the dendritic branching pattern and dendritic spine density of hippocampal CA1 pyramidal neurons between Dp(10)2Yey (n=27) and WT (n=18) mice. The paired mean differences for comparisons are shown in the Gardner-Altman estimation plot. Both groups are plotted on the left axis; the mean difference is plotted on a floating axis on the right as a bootstrap sampling distribution. The mean difference is depicted as a dot; the 95% confidence interval is indicated by the limits of the vertical error bar. Statistical analysis was performed using the permutation t-test (with 5000 shuffles). All statistical details are presented in Data S1.

A

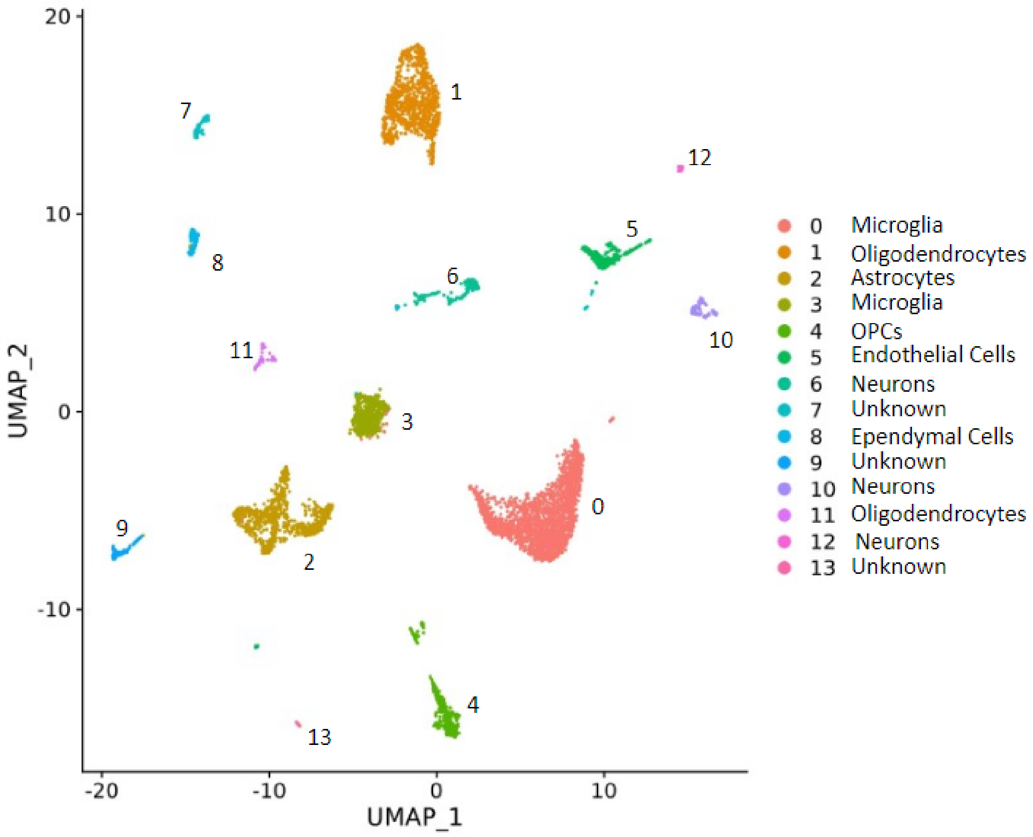

B

Cluster #	Cell Type	Cluster Markers	No. of Cells		Total No. of Cells	No. of DEGs
			Dp(10)2Yey	WT		(adjpval<0.01)
0	Microglia	Tmem119, Fosb, Rgs2, Klf4, Klf6, Ctss, C1qb, Hexb, C1qc, C1qa	1162	852	2014	576
1	Oligodendrocyte	Olig2, Olig1, Mbp, Mog, Sox10, Ermn, Plp1, Mal, Opalin, Trf, Mobp	529	440	969	99
2	Astrocytes	Gfap, Slc1a2, Slc1a3, Gja1, S100b, Aldoc, Clu, Cpe	543	346	889	205
3	Microglia	Ctss, C1qb, Hexb, C1qc, C1qa	317	241	558	9
4	Oligodendrocyte Precursor Cell	Pdgfra, Cacng4, Marcks, Olig1, Cspg4	219	163	382	141
5	Endothelial Cell	Bsg, Ly6c1, Igfbp7, Flt1, Ly6a	145	137	282	749
6	Neurons	Rtn1, Calm2, Tubb3	111	77	188	37
7	Unknown		83	50	133	4
8	Ependymal Cell	Calml4, Ak7, Ccdc153, 1110017D15Rik, DynlRb2	52	76	128	451
9	Unknown		74	51	125	1
10	Neurons	Atp1b1, Olfm1, Rtn1, Snap25, Calm2, Cck, Syp, Vamp2	54	65	119	0
11	Oligodendrocytes	Vcan, Cacng4, Marcks, Olig1, Plp1, Mbp	43	34	77	16
12	Neurons	Cck, Tubb3, Syp, Vamp2	26	25	51	1
13	Unknown		8	14	22	47
		Totals:	3366	2571	5937	2336

Figure S11: Characterization of hippocampal cell clusters from Dp(10)2Yey DS mice. **Related to STAR Methods, result session: Single-cell RNA sequencing of Dp(10)2Yey Hippocampus, and discussion.** A total of 5937 cells were sequenced from two experiments (Dp(10)2Yey, n=2; WT, n=2). t-SNE analysis was used to define cell clusters and UMAP to visualise them. 14 cell clusters were defined, 11 of which were identified by known markers of microglia, oligodendrocytes, astrocytes, oligodendrocyte precursor cells (OPCs), endothelial cells, neurons, and ependymal cells.

Table S1: Pearson's R and P-values for the correlations between integrated theta power and behavioural performance. **Related to figures 3 and 4.**

**Integrated theta power**				
	**Novel**		**Familiar**	
WT	Time in the periphery	Mobile time in the periphery	Time in the periphery	Mobile time in the periphery
Movement	R^2=0.85; p=0.003	R^2=0.72; p=0.015	R^2=0.25; p=0.254	R^2=0.43; p=0.110
Stationary	R^2=0.76; p=0.010	R^2=0.68; p=0.021	R^2=0.23; p=0.235	R^2=0.23; p=0.270
Dp(10)2Yey				
Movement	R^2=0.43; p=0.112	R^2=0.87; p=0.002	R^2=0.05; p=0.62	R^2=0.01; p=0.808
Stationary	R^2=0.28; p=0.224	R^2=0.72; p=0.016	R^2=0.16; p=0.37	R^2=0.10; p=0.488
**Phase-amplitude coupling (6–12-Hz theta phase and 60–120-Hz gamma amplitude)**				
	**Novel**		**Familiar**	
WT	Time in the periphery	Mobile time in the periphery	Time in the periphery	Mobile time in the periphery
Movement	R^2=0.87; p=0.048	R^2=0.66; p=0.027	R^2=0.08; p=0.536	R^2=0.24; p=0.269
Stationary	R^2=0.46; p=0.089	R^2=0.67; p=0.024	R^2=0.13; p=0.415	R^2=0.08; p=0.531
Dp(10)2Yey				
Movement	R^2=0.34; p=0.173	R^2=0.76; p=0.010	R^2=0.31; p=0.196	R^2=0.16; p=0.370
Stationary	R^2=0.04; p=0.663	R^2=0.16; p=0.369	R^2=0.57; p=0.050	R^2=0.28; p=0.222

Table S2: Comparing the cell density of Parvalbumin (PV) and neuropeptide Y (NPY) expressing interneurons in the hippocampus and the cell density of PV and calretinin (CR) - expressing interneurons in mPFC between Dp1Tyb DS mice and wild-type littermate controls. **Related to figures 5 and 6.** The effect sizes (mean difference) and CIs (95% confidence: lower limit and upper limit) are reported in this table. The p value(s) reported are the likelihood(s) of observing the effect size(s) if the null hypothesis of zero difference is true. For each permutation P value, 5000 shuffles of the control and test labels were performed. (DG= dentate gyrus, ACC= anterior cingulate cortex, IL= infralimbic area, PL= perlimbic area).

**Hippocampus**						
**PV-expressing interneurons**	N for Dp(10)2Yey	N for control	Mean difference	CI_lower_limit	CI_upper limit	P value permutation
Hippocampus-all	3	3	11.31	-298.84	223.11	0.89
CA1	3	3	-285.26	-1144.07	237.61	0.68
CA2	3	3	-360.70	-1024.05	302.64	0.39
CA3	3	3	30.33	-404.28	357.24	0.89
DG-molecular layer	3	3	-16.24	-149.78	73.40	0.94
DG-granule cell layer	3	3	289.72	-881.24	1315.85	0.63
DG-polymorphic layer	3	3	119.62	-390.23	578.90	0.60
Fasciola cinerea	3	3	301.69	-174.08	961.41	0.48
**NPY-expressing interneurons**	N for Dp(10)2Yey	N for control	Mean difference	CI_lower_limit	CI_upper limit	P value permutation
Hippocampus-all	3	3	258.06	-358.19	690.91	0.49
CA1	3	3	55.3	-163.57	304.30	0.70
CA2	3	3	-110.62	-372.87	76.46	0.53
CA3	3	3	29.81	-169.03	284.91	0.80
DG-molecular layer	3	3	408.37	-132.05	803.91	0.14
DG-granule cell layer	3	3	692.97	-180.56	1466.24	0.20
DG-polymorphic layer	3	3	235.91	-640.56	779.04	0.42
Fasciola cinerea	3	3	494.61	-1741.85	1868.38	0.63
**mPFC**						
**PV-expressing interneurons**	N for Dp(10)2Yey	N for control	Mean difference	CI_lower_limit	CI_upper limit	P value permutation
mPFC-all	3	3	-247.99	-1100.33	628.19	0.60
ACC	3	3	-303.25	-1913.15	1306.65	0.49
IR	3	3	-125.68	-640.84	562.96	0.70
PL	3	3	-315.04	-1458.85	828.76	0.40
**CR-expressing interneurons**	N for Dp(10)2Yey	N for control	Mean difference	CI_lower_limit	CI_upper limit	P value permutation

mPFC-all	3	4	266.46	-170.15	698.29	0.38
ACC	3	4	29.52	-563.32	408.89	0.94
IR	3	4	742.52	107.02	1320.30	0.08
PL	3	4	27.33	-465.48	482.83	0.91
**Hippocampus**						
**PV-expressing interneurons**	N for Dp(10)2Yey	N for control	Mean difference	CI_lower_ limit	CI_upper limit	P value permutation
Hippocampus -all	3	3	11.31	-298.84	223.11	0.89
CA1	3	3	-285.26	-1144.07	237.61	0.68
CA2	3	3	-360.70	-1024.05	302.64	0.39
CA3	3	3	30.33	-404.28	357.24	0.89
DG-molecular layer	3	3	-16.24	-149.78	73.40	0.94
DG-granule cell layer	3	3	289.72	-881.24	1315.85	0.63
DG-polymorphic layer	3	3	119.62	-390.23	578.90	0.60
Fasciola cinerea	3	3	301.69	-174.08	961.41	0.48
**mPFC**						
**PV-expressing interneurons**	N for Dp(10)2Yey	N for control	Mean difference	CI_lower_ limit	CI_upper limit	P value permutation
mPFC-all	3	3	-247.99	-1100.33	628.19	0.60
ACC	3	3	-303.25	-1913.15	1306.65	0.49
IR	3	3	-125.68	-640.84	562.96	0.70
PL	3	3	-315.04	-1458.85	828.76	0.40
**CR-expressing interneurons**	N for Dp(10)2Yey	N for control	Mean difference	CI_lower_ limit	CI_upper limit	P value permutation
mPFC-all	3	4	266.46	-170.15	698.29	0.38
ACC	3	4	29.52	-563.32	408.89	0.94
IR	3	4	742.52	107.02	1320.30	0.08
PL	3	4	27.33	-465.48	482.83	0.91

Table S3: GSEA of Pathways Significantly Upregulated in male Dp(10)2Yey HPC. **Related to STAR Methods, result session: Single-cell RNA sequencing of Dp(10)2Yey Hippocampus, and discussion.** Gene Set Enrichment Analysis (GSEA) was used to determine whether a defined set of genes showed statistically significant concordant differences between Dp(10)2Yey and WT cells. In total 60 Hallmark pathways were upregulated in the Dp(10)2Yey cells compared to WT cells (FWER < 0.05). [1]NES is calculated as described in Methods. [2]adjusted p value is the significance of pathway enrichment as described in Methods, pathways are ordered by NES.

Cell Cluster	Cell Cluster ID	Hallmark Pathway	NES [1]	adjusted p-value[2]
0	Microglia	HALLMARK COAGULATION	1.92	0.006
		HALLMARK EPITHELIAL MESENCHYMAL TRANSITION	1.77	0.006
		HALLMARK COMPLEMENT	1.73	0.006
		HALLMARK MYOGENESIS	1.69	0.006
		HALLMARK TNFA SIGNALING VIA NFKB	1.69	0.006
		HALLMARK ALLOGRAFT REJECTION	1.61	0.006
		HALLMARK P53 PATHWAY	1.52	0.006
		HALLMARK APOPTOSIS	1.50	0.011
		HALLMARK UV RESPONSE UP	1.50	0.010
		HALLMARK INTERFERON GAMMA RESPONSE	1.44	0.011
		HALLMARK MTORC1 SIGNALING	1.43	0.010
		HALLMARK HYPOXIA	1.40	0.032
		HALLMARK APICAL JUNCTION	1.39	0.045
		HALLMARK MYC TARGETS V1	1.39	0.011
		HALLMARK OXIDATIVE PHOSPHORYLATION	1.32	0.021
1	Oligodendrocytes	HALLMARK MYOGENESIS	1.49	0.011
		HALLMARK COMPLEMENT	1.45	0.011
		HALLMARK ADIPOGENESIS	1.40	0.011
		HALLMARK OXIDATIVE PHOSPHORYLATION	1.39	0.011
		HALLMARK UV RESPONSE UP	1.37	0.025
		HALLMARK APOPTOSIS	1.35	0.033
		HALLMARK MTORC1 SIGNALING	1.30	0.025
		HALLMARK MYC TARGETS V1	1.26	0.025
2	Astrocytes	HALLMARK COMPLEMENT	1.93	0.020
3	Microglia	HALLMARK OXIDATIVE PHOSPHORYLATION	2.37	0.043
		HALLMARK COMPLEMENT	1.87	0.043
		HALLMARK ADIPOGENESIS	1.74	0.043
4	OPCs	HALLMARK COAGULATION	1.71	0.016
		HALLMARK OXIDATIVE PHOSPHORYLATION	1.70	0.016
		HALLMARK MYC TARGETS V1	1.60	0.016
		HALLMARK EPITHELIAL MESENCHYMAL TRANSITION	1.46	0.034
		HALLMARK G2M CHECKPOINT	1.40	0.034

		HALLMARK UV RESPONSE UP	1.39	0.034
		HALLMARK ADIPOGENESIS	1.34	0.034
5	Endothelial Cells	HALLMARK COMPLEMENT	2.05	0.004
		HALLMARK COAGULATION	2.04	0.004
		HALLMARK TNFA SIGNALING VIA NFKB	2.01	0.004
		HALLMARK ALLOGRAFT REJECTION	2.00	0.004
		HALLMARK EPITHELIAL MESENCHYMAL TRANSITION	1.93	0.004
		HALLMARK UV RESPONSE UP	1.79	0.004
		HALLMARK HYPOXIA	1.75	0.004
		HALLMARK CHOLESTEROL HOMEOSTASIS	1.74	0.027
		HALLMARK P53 PATHWAY	1.66	0.004
		HALLMARK MTORC1 SIGNALING	1.61	0.004
		HALLMARK INTERFERON GAMMA RESPONSE	1.60	0.004
		HALLMARK APICAL JUNCTION	1.59	0.004
		HALLMARK MYOGENESIS	1.59	0.034
		HALLMARK OXIDATIVE PHOSPHORYLATION	1.54	0.011
		HALLMARK KRAS SIGNALING UP	1.50	0.034
		HALLMARK APOPTOSIS	1.49	0.027
		HALLMARK INFLAMMATORY RESPONSE	1.48	0.027
6	Neurons	HALLMARK COMPLEMENT	1.73	0.047
		HALLMARK REACTIVE OXYGEN SPECIES PATHWAY	1.68	0.047
		HALLMARK OXIDATIVE PHOSPHORYLATION	1.53	0.047
8	Ependymal Cells	HALLMARK OXIDATIVE PHOSPHORYLATION	2.31	0.022
		HALLMARK MYC TARGETS V1	2.22	0.022
		HALLMARK ADIPOGENESIS	1.53	0.030
11	Oligodendrocytes	HALLMARK OXIDATIVE PHOSPHORYLATION	1.87	0.036
		HALLMARK MYC TARGETS V1	1.38	0.036
12	Neurons	HALLMARK MYOGENESIS	1.62	0.039
